# Identification and validation of SQLE in steroid-induced osteonecrosis of the femoral head: a bioinformatics and experimental study

**DOI:** 10.1186/s13018-025-06305-x

**Published:** 2025-10-17

**Authors:** Yixiang He, Wanjia Qiao, Kui Zhang, Xuewen Shi, Wenji Wang

**Affiliations:** 1 The First Clinical Medical College of Lanzhou University, Lanzhou, 730000 Gansu China; 2 Department of Orthopedics, The First Hospital of Lanzhou University, Lanzhou, 730000 Gansu China

**Keywords:** Osteonecrosis of the femoral head, Lipid metabolism, Bioinformatics, Transcriptome sequencing, Squalene epoxidase, Terbinafine

## Abstract

**Background:**

Osteonecrosis of the femoral head (ONFH) is a prevalent and refractory hip disease. In this study, we investigated the expression profiles of lipid metabolism-related genes in ONFH and evaluated the potential therapeutic effects of squalene epoxidase (SQLE) and its inhibitors.

**Methods:**

Dexamethasone was used to establish an in vitro ONFH model in MC3T3-E1 cells. Differentially expressed genes (DEGs) in the model group were identified through transcriptome sequencing. Lipid metabolism-related DEGs were extracted from GeneCards, and hub genes were determined via a protein–protein interaction (PPI) network. The expression patterns and diagnostic value of these hub genes were further validated using the GEO dataset. qRT-PCR and WB were performed to detect the expression of hub genes. Subsequently, the effects of SQLE knockdown and overexpression on osteoblast proliferation, apoptosis, and osteogenic differentiation were further evaluated. The involvement of ferroptosis was assessed by measuring Fe^2^⁺, ROS, MDA and GSH levels, with or without the ferroptosis inhibitor Fer-1. An in vivo rat model of ONFH was established. The therapeutic effects of SQLE inhibitors were evaluated by micro-CT, H&E staining, immunohistochemistry, serum lipid profiles, and ferroptosis-related indices.

**Results:**

A total of 579 DEGs were identified, and these DEGs were enriched in various functions and pathways. After constructing the PPI network, five hub genes (*Fdps, Lss, Sqle, Nsdhl,* and *Hmgcs2*) were identified. GEO dataset validation confirmed consistent expression trends and diagnostic value for these genes. In vitro, SQLE knockdown significantly alleviated MC3T3-E1 damage, and mitigated ferroptosis-related oxidative stress. Conversely, SQLE overexpression aggravated these effects. In vivo, terbinafine treatment improved bone microarchitecture, reduced empty lacunae, normalized serum lipid profiles, and suppressed ferroptosis markers in ONFH rats.

**Conclusions:**

This study reveals the role of SQLE in ONFH. Targeting SQLE, either through genetic silencing or pharmacological inhibition, alleviates osteoblast dysfunction and bone loss, providing a potential therapeutic strategy for ONFH.

**Supplementary Information:**

The online version contains supplementary material available at 10.1186/s13018-025-06305-x.

## Introduction

Osteonecrosis of the femoral head (ONFH), also known as avascular necrosis of the femoral head (ANFH), is a prevalent and refractory disease of the hip joint in clinical practice, leading to high disability rates [1, 2]. With the widespread use of glucocorticoids (GCs) in clinical, the incidence of ONFH has been gradually increasing in recent years. Long-term use of GCs has been identified as the primary factor for ONFH, with GC-induced ONFH accounting for over 50% of non-traumatic patients in some studies [3–5]. It is characterized by compromised blood circulation within the femoral head, ischemic changes in the major components of the femoral head including osteocytes and bone marrow, followed by cellular necrosis and apoptosis, ultimately leading to structural modifications of the femoral head. In the advanced stage, most patients may experience collapse of the femoral head, leading to severe hip dysfunction that often requires surgical intervention and significantly impacts patients' quality of life [6–10]. Furthermore, considering the higher incidence of ONFH in individuals under the age of 50, it significantly increases the probability of patients undergoing revision surgery, thereby imposing a substantial burden on both patients and society [11–13].

The pathogenesis of ONFH involves an interplay of multifaceted factors and mechanisms, including hereditary susceptibility, lipid metabolism disorders, fat embolism, coagulation dysfunction, endothelial injury, osteocyte apoptosis, increased intraosseous pressure in the femoral head, dysregulation of the immune system, and other factors [14–18]. Lipid metabolism is a critical physiological process essential for maintaining bodily functions. In recent years, with the advancement of research on ONFH, accumulating evidence suggests that lipid metabolic disorders significantly contribute to the pathogenesis of ONFH [14, 19–21]. Especially in patients with long-term glucocorticoid use and alcohol abuse, lipid metabolism disorder has been regarded as an important risk factor for the onset of ONFH. GCs can cause disorders in lipid metabolism, leading to the accumulation of adipocytes in the bone marrow, increased intra-medullary pressure, compromised arterial blood supply, and lipid deposition within osteocytes. These effects can induce the differentiation of pre-osteocytes into adipocytes, ultimately resulting in subchondral bone degeneration, necrosis, and subsequent collapse of the femoral head [22–24]. Abnormal lipid metabolism can also induce vascular endothelial injury, aggravate vascular inflammatory responses, and ultimately result in femoral head ischemia and bone cell death [25]. Therefore, an in-depth investigation of lipid metabolism disorders in ONFH is crucial for elucidating disease mechanisms, identifying effective therapeutic targets, and developing novel therapeutic strategies.

With the advancement of high-throughput sequencing technologies, transcriptome sequencing and microarrays analysis have emerged as robust tools for investigating disease regulatory mechanisms. This study aims to investigate alterations in the expression of lipid metabolism-related genes in ONFH using transcriptome sequencing and bioinformatics analysis, and to identify key differentially expressed genes and their associated regulatory pathways. The role of hub genes will be verified through subsequent experimental research. This research elucidates the potential role of lipid metabolism-related gene SQLE in the development of steroid-induced femoral head necrosis, thereby providing a theoretical foundation for targeted clinical treatments.

## Materials and methods

### Cell culture and treatment

MC3T3-E1 cells were purchased from Procell Life Science & Technology Co., Ltd (Wuhan, China) and cultured in α-MEM (GIBCO, USA) containing 10% fetal bovine serum (Biological Industries, Israel) and 1% penicillin/streptomycin (Biological Industries, Israel) at 37 °C with 5% CO_2_. Cells were treated with dexamethasone (DEX, Yuanye Biotechnology, China) or Ferrostatin-1 (Fer-1, MedChemExpress, USA) at various concentrations. The control group was treated with medium containing 0.1% dimethyl sulfoxide (DMSO, Solarbio, China).

### Cell viability

Cell viability was assessed using the CCK-8 kit (APExBIO, USA). MC3T3-E1 cells were inoculated into 96-well plates (4000 cells per well). After treatment, 10 μL of CCK-8 reagent was added to each well, and the cells were incubated for 2 h. The absorbance at 450 nm was measured using the Synergy H1 plate reader (BioTek, USA).

### Flow cytometry

Cell apoptosis was detected using the Annexin V-FITC/PI apoptosis kit (Elabscience, China). MC3T3-E1 cells were inoculated into 12-well plates (1 × 10^5^ cells per well). After collecting the cells, they were resuspended in 500 μL of Binding Buffer containing 5 μL of Annexin V-FITC and 5 μL of PI reagent. Finally, the total apoptosis rate was analyzed by flow cytometry (Agilent, USA).

Cell cycle was detected using the Cell Cycle Assay Kit (Elabscience, China). MC3T3-E1 cells were harvested and resuspended in PBS. Subsequently, pre-cooled ethanol was added to the cells, which were fixed at − 20 °C overnight. The next day, after centrifugation, RNase A solution and PI staining solution (50 μg/mL) were added for incubation, and the cell cycle was analyzed by flow cytometry.

### Alkaline phosphatase (ALP) Staining

ALP staining was performed using BCIP/NBT alkaline phosphatase staining solution (Beyotime Biotechnology, China). The cells were treated with osteogenic differentiation medium (containing 10% FBS, 1% P/S, 10 mM β-sodium glycerophosphate, 50 μg/mL vitamin C, and 10^− 8^ mol/L dexamethasone). After 14 days, the cells were fixed with 4% paraformaldehyde. ALP staining solution was added, and the cells were incubated at room temperature and observed under the microscope (Olympus, Japan).

### Transcriptome sequencing and enrichment analysis

MC3T3-E1 cells were inoculated into 100 mm dishes (1 × 10^6^ cells per dish). After dexamethasone treatment, total RNA was extracted from both the control and ONFH cell model groups for downstream transcriptome sequencing. RNA quality was assessed using the Agilent 5300 Bioanalyzer (Agilent, USA), and RNA concentration was measured using the NanoDrop 2000 (Thermo Fisher Scientific, USA). High-quality RNA samples (OD_260/280_ = 1.8–2.2, OD_260/230_ ≥ 2.0, RQN ≥ 6.5) were selected for subsequent library construction.

RNA purification, reverse transcription, library construction and sequencing were performed at Shanghai Majorbio Bio-pharm Biotechnology Co., Ltd. (Shanghai, China). Sequencing libraries were prepared following Illumina® Stranded mRNA Prep, Ligation (Illumina, USA). Shortly, messenger RNA was isolated according to polyA selection method by oligo(dT) beads and then fragmented by fragmentation buffer firstly. Secondly double-stranded cDNA was synthesized using a SuperScript double-stranded cDNA synthesis kit (Invitrogen, USA) with random hexamer primers. Then the synthesized cDNA was subjected to end-repair, phosphorylation and adapter addition according to library construction protocol. Libraries were size selected for cDNA target fragments of 300 bp on 2% Low Range Ultra Agarose followed by PCR amplified using Phusion DNA polymerase (NEB) for 15 PCR cycles. After quantified by Qubit 4.0, the sequencing library was performed on NovaSeq X Plus platform (PE150) using NovaSeq Reagent Kit (Illumina, USA). Each sample can generate more than 6.4 Gb of clean data.

Raw sequencing reads were subjected to quality control using Fastp (https://github.com/OpenGene/fastp, version 0.23.4) [26] to remove adapter sequences, low-quality bases (Phred score < 20), and reads with more than 10% uncertain nucleotides, and reads with lengths shorter than 20 bp. The clean reads were aligned to the mouse reference genome (GRCm39, http://asia.ensembl.org/Mus_musculus/Info/Index) using HISAT2 (http://ccb.jhu.edu/software/hisat2/index.shtml, version 2.2.1) [27] with default parameters. Gene-level quantification was then performed using FeatureCounts (http://subread.sourceforge.net/featureCounts.html, version 2.0.3) [28] against the GENCODE M33 annotation. These steps ensured high mapping efficiency and accurate read counting for differential expression analysis.

Differential expression analysis was performed on the generated read count matrix using DESeq2 (version 1.42.0) to identify differentially expressed genes (DEGs) [29]. Genes were considered significantly differentially expressed if they met the following criteria: log_2_(fold change) ≥ 1 for up-regulated genes and log_2_(fold change) ≤ − 1 for down-regulated genes, combined with *P*-adjust ≤ 0.05. GO functional enrichment and KEGG pathway analyses were carried out using Goatools (https://github.com/tanghaibao/GOatools, version 1.4.4) and SciPy package (https://scipy.org/install/, version 1.7.3), respectively. These analyses were supported by the Majorbio Cloud Platform (https://cloud.majorbio.com/, 2024 Release) [30]. A pathway was considered significantly enriched when the *P*-value was less than 0.05.

### Screening of lipid metabolism-related genes

A list of lipid metabolism-related genes was retrieved using the keyword "lipid metabolism" from GeneCards (https://www.genecards.org/) [31]. Genes with a relevance score > 3 were selected, and the DEGs were compared with these lipid metabolism-related genes to identify intersecting genes by constructing a Venn diagram.

### Protein–protein interaction (PPI) network

These genes were entered into the STRING database (Search Tool for the Retrieval of Interacting Genes/Proteins, http://string-db.org/)[32]. Correlation pairs with a combined score > 0.4 were selected to construct a PPI network. Visualization was performed using Cytoscape software (version 9.3.1, http://www.cytoscape.org/)[33]. The MCODE (Molecular Complex Detection) plugin and CytoHubba were used to identify key molecular modules.

### Receiver operating characteristic (ROC) curve and gene set enrichment analysis (GSEA) analysis

The validation dataset was obtained from the Gene Expression Omnibus (GEO) database (https://www.ncbi.nlm.nih.gov/geo/) [34]. As this validation dataset was generated using microarray technology, which differs from the count-based RNA-seq data analyzed by DESeq2 in our primary cohort, the "limma" package in R was used to perform differential expression analysis [35]. The "ggplot2" package was employed to draw violin plots and ROC curves for the expression of each hub gene. The area under the ROC curve (AUC) was calculated to assess the sensitivity and specificity of each gene in distinguishing ONFH samples from controls. An AUC value close to 1 indicates high diagnostic accuracy. Then, to understand the potential biological functions and signaling pathways of hub genes in ONFH, GSEA was conducted using the "clusterProfiler" package on the ranked gene list derived from the limma analysis.

### Regulatory network

The Drug-Gene Interaction Database (DGIdb, https://dgidb.org/)[36] is a comprehensive database designed to provide detailed information on interactions between drugs and genes. In this study, we retrieved therapeutic drugs and potential targets for the hub genes from DGIdb and constructed a drug-gene interaction network.

The upstream miRNAs and lncRNAs with high binding affinity to each hub gene were predicted using the miRanda (http://www.microrna.org/) [37], miRDB (https://mirdb.org/) [38], and TargetScan (https://www.targetscan.org/) [39] databases. Then, a ceRNA network (lncRNA-miRNA-mRNA) was constructed and subsequently visualized.

### Cell transfection

The siRNAs targeting SQLE were designed and synthesized by GenePharma (Shanghai, China). The plasmids targeting SQLE were provided by Vigene Biosciences (Shandong, China). siRNA and overexpression plasmid, along with corresponding negative controls, were transfected into the cells using jetPRIME transfection reagent (Polyplus-transfection, France), following the manufacturer's protocol. The siRNA sequences are provided in Table S1.

### 5-ethynyl-2′-deoxyuridine (EdU) assay

EdU assay was performed with BeyoClick™ EdU Cell Proliferation Kit with Alexa Fluor 594 (Beyotime, China). The experiment was carried out strictly following the manufacturer's instructions. Samples were observed with the microscope. The percentage of Edu-positive cells was calculated as the number of Edu-positive cells out of the total number of cells.

### Quantitative real-time PCR (qRT-PCR)

Total RNA was extracted with TRIzol (Servicebio, China). Subsequently, the M5 Sprint qPCR RT kit with gDNA remover (Mei5bio, China) was used to reverse transcribe the RNA into cDNA. Quantitative real-time PCR was carried out using an RT-PCR amplifier system (Bio-Rad, USA) and the M5 HiPer SYBR Premix EsTaq (Mei5bio, China). The results were normalized using *Gapdh*, and the relative expression levels of each gene were calculated using the 2^−ΔΔCT^ method. PCR primer synthesis was performed by Tsingke Biotech Co., Ltd (Xi'an, China). The primer sequences are listed in Table 1.

**Table 1:** The information of the primers’ sequencing

Gene	Forward (5ʹ to 3ʹ)	Reverse (5ʹ to 3ʹ)
*Nsdhl*	CGTCCTCATGGCATTTTCGG	TCAGCGGCTAAGATGTGTCC
*Fdps*	GCAGAGTTCCTATCAGACAG	CATTGGCGTGTTCCTTCT
*Hmgcs2*	TGTTCAACCAGAAGACCAA	TCTCATCCACTCGTTCAAG
*Lss*	TTCGTTGGTCAGTGGATG	CCTCTAACAGTGCTTGGATA
*Sqle*	TGTTGCGGATGGACTCTTCT	GAGAACTGGACTGGGGTTGA
*Opn*	AATGCTGTGTCCTCTGAAG	ATCGTCATCATCATCGTCAT
*Ocn*	AACGCATCTATGGTATCACT	GCACTTCCTCATCTGAACT
*Runx2*	AATGCCTCCGCTGTTATG	CTTCTGTCTGTGCCTTCTT
*Gapdh*	TCTCCTGCGACTTCAACA	TGTAGCCGTATTCATTGTCA

### Western blot (WB)

Cells were lysed with RIPA buffer (Boster, China), and the protein concentration of the samples was determined using a BCA protein assay kit (Boster, China). Subsequently, 30 μg of total protein was separated by electrophoresis on an 8% SDS-PAGE and then transferred to a PVDF membrane (Millipore, USA). A marker (Servicebio, China) was used to determine the molecular weight of the protein bands. After blocking, membranes were incubated overnight at 4 °C with the following primary antibodies: anti-SQLE (1:1000, Proteintech, China), anti-OPN (1:600, Servicebio, China), anti-Collagen I (1:600, Servicebio, China), anti-RUNX2 (1:600, Servicebio, China), anti-Flag (1:2000, Proteintech, China), and anti-GAPDH (1:10,000, Proteintech, China). The next day, the membrane was rinsed and then incubated with the secondary antibody (1:10,000, Proteintech, China) at room temperature for 1 h. After washing, the protein bands were visualized using an ECL kit (Boster, China), and the images were captured using an automated gel imaging system (Clinx, China). GAPDH was used as the internal reference protein for normalization of the results.

### Animal treatment

This experiment utilized 6-week-old male SPF-grade Sprague–Dawley (SD) rats weighing 200–220 g, provided by the Laboratory Animal Center of Lanzhou University. All animals were housed under specific pathogen-free conditions at 22 ± 1 °C and 60 ± 10% humidity, with free access to food and water. All procedures were approved by the Ethics Committee of the Laboratory Animal Center of Lanzhou University (Approval Number: MECI20250005).

All animals were acclimatized for one week and then randomly divided into the control group, model group, and treatment group, with six rats in each group. The model was established following a previously described protocol [40]. On days 1 and 2, rats in the model and treatment groups received intraperitoneal injections of lipopolysaccharide (LPS, Sigma-Aldrich, USA) at 20 μg/kg/day. From days 3 to 5, both groups were given intramuscular injections of methylprednisolone (MPS, Sinopharm, China) at 40 mg/kg/day. Additionally, the treatment group received oral administration of the SQLE inhibitor terbinafine (TB, MedChemExpress, USA) at 80 mg/kg/day, beginning on the day of model induction, following previously published studies [41]. The control group received an equivalent volume of saline orally and by injection. After eight weeks, bilateral femoral head tissues and serum samples were collected for analysis.

### Micro-CT

At the endpoint, the femoral heads of the rats were scanned using a micro-CT system (Pingseng Scientific, China). The region of interest (ROI) was analyzed using 3D reconstruction and data analysis software. Structural parameters, including bone volume/total volume (BV/TV), trabecular thickness (Tb.Th), trabecular separation (Tb.Sp), and bone mineral density (BMD), were analyzed.

### H&E staining

The bone tissue samples from rats were fixed in a 4% paraformaldehyde solution, subsequently decalcified, and embedded in paraffin wax. The embedded bone specimens were sectioned into 5-µm-thick slices along the sagittal plane using a microtome. These sections were then stained with hematoxylin and eosin (H&E) following standard histological protocols to examine the structure of the femoral head. The stained sections were analyzed under a microscope.

### Immunohistochemical (IHC) Staining

The sections of the femoral head underwent defatting, rehydration, and antigen retrieval. They were then blocked with Tris-buffered saline containing 1% bovine serum albumin for 2 h at room temperature. Subsequently, the sections were incubated sequentially with anti-SQLE (1:200), anti-Collagen I (1:200), a secondary antibody (1:500), and DAB detection solution. After color development, the sections were examined under a microscope.

### Detection of reactive oxygen species (ROS)

The level of ROS in MC3T3-E1 cells was detected using the Reactive Oxygen Species (ROS) Fluorometric Assay Kit (Green) (Elabscience, China). In brief, the cells were incubated with the fluorescent probe dichlorofluorescein diacetate (DCFH-DA) at a concentration of 10 μmol/L for 1 h at 37 °C, followed by three washes with serum-free medium to remove excess DCFH-DA. Fluorescence was then visualized using a fluorescence microscope. Additionally, the treated cells were collected, resuspended in PBS, and analyzed by flow cytometry to quantify fluorescence intensity.

### Detection of the Fe^2+^

The Fe^2+^ levels in MC3T3-E1 cells and serum were determined using a Ferric and Ferrous Ion Assay Kit, Colorimetric (Beyotime, China). The cells were lysed with BeyoLysis™ Buffer H for Metabolic Assay at 4 °C for 10 min and subsequently centrifuged at 12,000 × g for 15 min. The collected cell lysates or serum samples were subjected to acid treatment, and reagents were added sequentially following the manufacturer's instructions. The reaction mixture was incubated at 37 °C for 30 min, after which the absorbance at 593 nm was measured.

### Detection of the malondialdehyde (MDA)

The MDA content in MC3T3-E1 cells and serum was determined using the Lipid Peroxidation MDA Assay Kit (Beyotime, China). Cellular proteins were extracted using RIPA buffer and quantified with the BCA Protein Assay Kit. After the MDA reaction reagent was added to the protein samples or serum, the mixtures were incubated at 100 °C for 15 min and then centrifuged at 1000 × g for 15 min. The absorbance at 532 nm was subsequently measured using a microplate reader.

### Detection of glutathione (GSH) and oxidized glutathione (GSSG)

The levels of GSH and GSSG were determined using the Total Glutathione (T-GSH)/Oxidized Glutathione (GSSG) Colorimetric Assay Kit (Elabscience, China). For cell samples, 10^6^ cells were lysed with 400 μL of lysis buffer and then centrifuged at 4 °C and 10,000 × g for 10 min. Serum samples were tested directly. For the detection of total GSH, total glutathione detection solution was added to the lysate and incubated at room temperature for 25 min, and the optical density was measured at 412 nm. For the detection of GSSG, the lysate was first incubated with GSH scavenger auxiliary solution and then reacted with total glutathione detection solution. The contents of total glutathione and GSSG were calculated based on the standard curve. The GSH concentration was calculated using the following formula: GSH concentration = total glutathione concentration—2 × GSSG concentration.

### Lipid level detection

For serum samples, the lipid levels were detected using Total cholesterol assay kit, Triglyceride assay kit, Low-density lipoprotein cholesterol assay kit, and High-density lipoprotein cholesterol assay kit (Nanjing Jiancheng Bioengineering Institute, China). The serum of rats was thawed on ice and mixed. Then, the levels of total cholesterol (TC), triglyceride (TG), high-density lipoprotein cholesterol (HDL-C), and low-density lipoprotein cholesterol (LDL-C) in each group were detected using a microplate reader according to the instructions of the corresponding assay kits.

### Statistical analysis

All experiments were conducted at least three times. Data are presented as mean ± standard deviation (Mean ± SD). An independent samples t-test was used to compare data between two groups, while a one-way analysis of variance (ANOVA) was utilized for comparisons among multiple groups, followed by Tukey’s post hoc test. Statistical analyses were performed using Prism 9.5 software (GraphPad, USA). P values less than 0.05 were considered statistically significant.

## Results

### Establishment and validation of the cell model of ONFH

**Fig. 1:**
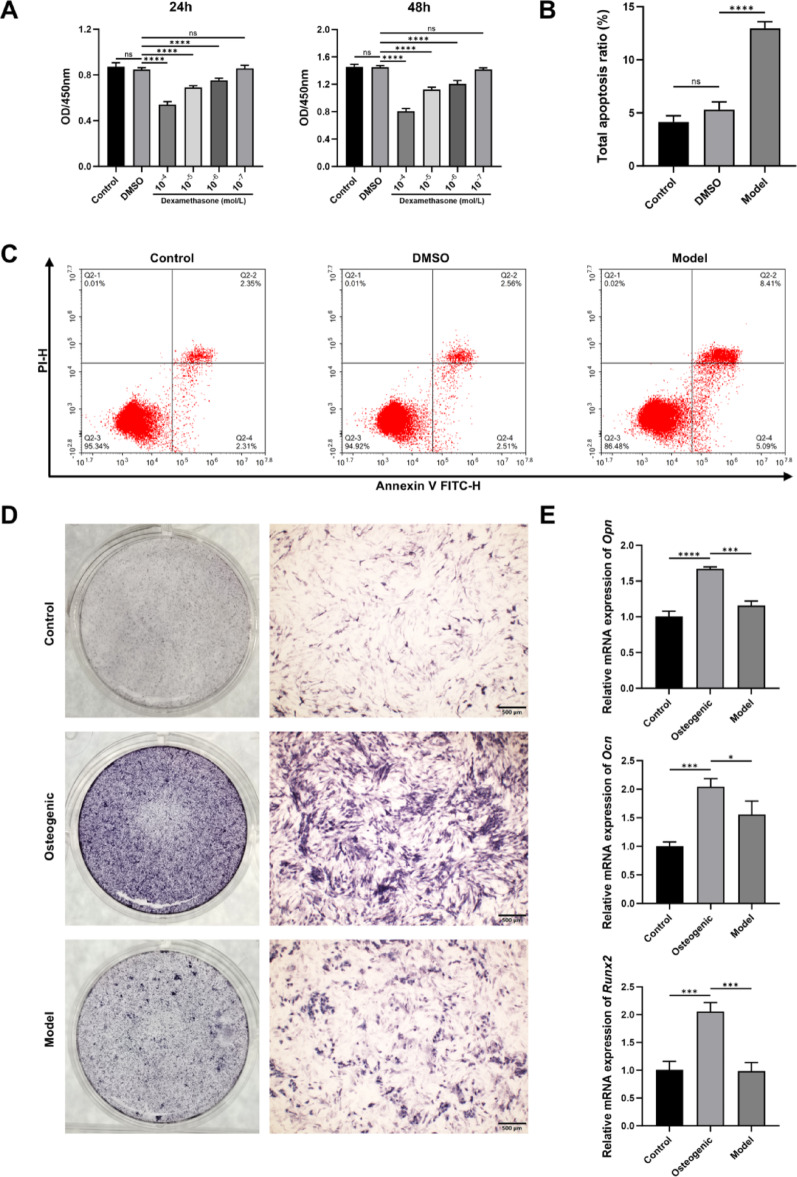
Establishment and validation of a cell model of ONFH. (**A**. CCK-8 detects cell viability. **B–C**. The apoptosis level. **D**. ALP staining. **E**. The mRNA expression of *Opn, Ocn,* and *Runx2*. n = 3. *P < 0.05, **P < 0.01, ***P < 0.001, ***P < 0.0001, ns means no significance. Scale bars = 500 μm)

To establish an in vitro model of glucocorticoid-induced ONFH, MC3T3-E1 cells were treated with different concentrations of dexamethasone (DEX), and the results of cell viability are shown in Fig. 1A. The results demonstrated that DEX suppressed cell viability in a dose-dependent manner (*P* < 0.001). A concentration of 10^− 5^ mol/L dexamethasone was selected for subsequent experiments. After treatment with dexamethasone, the apoptosis rate of the cells significantly increased (*P* < 0.001), as shown in Fig. 1B and C.

To investigate the effect of dexamethasone on osteogenic differentiation, cells were cultured in osteogenic induction medium in the presence or absence of DEX. ALP staining and qRT-PCR results indicated that the ALP staining intensity in the model group was markedly reduced, and the gene expressions of *Opn, Ocn,* and *Runx2* were significantly downregulated (*P* < 0.05), as shown in Fig. 1D and E. These results suggest that DEX can effectively induce apoptosis of MC3T3-E1 cells and inhibit their osteogenic differentiation, supporting the successful establishment of an in vitro ONFH model.

### Transcriptome sequencing and enrichment analysis

**Fig. 2:**
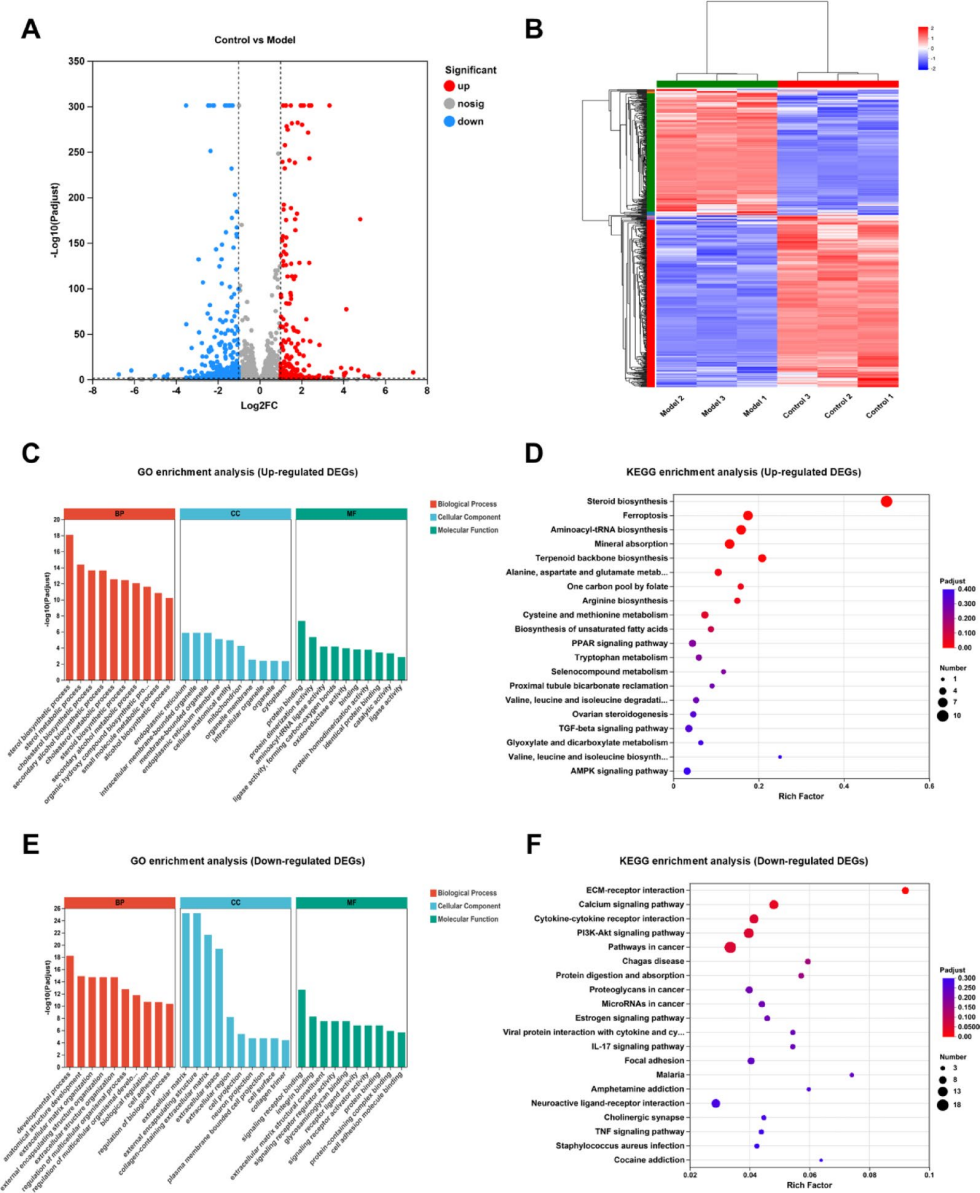
Identification of DEGs and enrichment analysis. (**A**. Volcano plot. **B**. Heatmap. **C**. Bar chart of GO enrichment of up-regulated DEGs. **D**. Bubble chart of KEGG enrichment results of up-regulated DEGs. **E**. Bar chart of GO enrichment of down-regulated DEGs. **F**. Bubble chart of KEGG enrichment results of down-regulated DEGs)

Transcriptome sequencing revealed significant changes in the gene expression profiles of MC3T3-E1 after dexamethasone treatment, identifying 579 DEGs compared to the control group, with 243 genes upregulated and 336 genes downregulated. The volcano plot and heatmap are shown in Figure 2A and B.

Figure 2C–F respectively present the KEGG pathway enrichment analysis and GO enrichment analysis results (including biological process, cellular component and molecular function) of up-regulated and down-regulated DEGs. The specific information is shown in Table S2. Functional enrichment analysis revealed that the up- and down-regulated DEGs were involved in distinct biological processes. The up-regulated DEGs were significantly enriched in biological processes (BP) such as sterol biosynthetic process, sterol metabolic process, and cholesterol biosynthetic process; in cellular components (CC) including endoplasmic reticulum, intracellular membrane-bounded organelle, and membrane-bounded organelle; and in molecular functions (MF) such as protein binding, protein dimerization activity, and aminoacyl-tRNA ligase activity. KEGG pathway analysis further indicated their involvement in Steroid biosynthesis, Ferroptosis, and Aminoacyl-tRNA biosynthesis, among others (Fig. 2C, D). In contrast, the down-regulated DEGs were enriched in biological processes related to developmental process, anatomical structure development, and extracellular matrix organization; in cellular components including extracellular matrix, external encapsulating structure, and collagen-containing extracellular matrix; and in molecular functions such as signaling receptor binding, integrin binding, and extracellular matrix structural constituent. KEGG pathway analysis showed significant enrichment in ECM–receptor interaction, Calcium signaling pathway, and Cytokine–cytokine receptor interaction (Fig. 2E, F).

### Identification of lipid metabolism-related hub genes

**Fig. 3:**
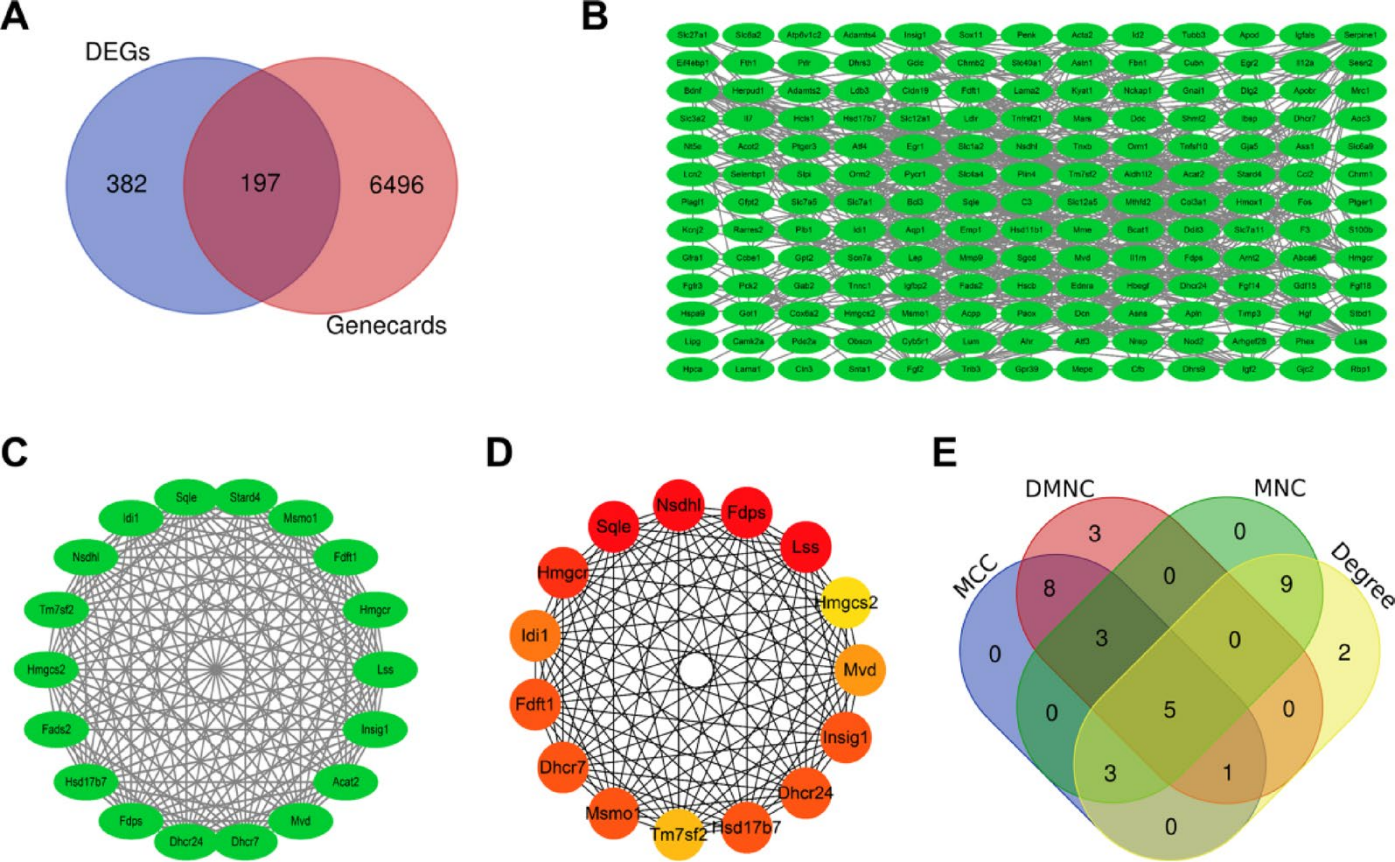
Identification of Lipid Metabolism-Related Hub Genes. (**A**. Venn diagram. **B**. PPI network. **C**. Key modules identified using MCODE. **D**. The MCC screens for the top 15 genes in Cytohubba. **E**. Identification of hub genes)

To investigate the potential involvement of lipid metabolism in ONFH, a total of 6,693 lipid metabolism-related genes were screened from the GeneCards database. After intersecting with DEGs, 197 genes were obtained, as shown in Fig. 3A. The STRING database was analyzed, and a PPI network consisting of 169 nodes and 654 edges was constructed (Fig. 3B). These nodes were subsequently imported into Cytoscape for further analysis. MCODE was utilized to identify relevant modules, which were then evaluated and ranked based on their significance within the entire network. The subnetwork with the highest score was selected, as shown in Fig. 3C. The entire network was comprehensively analyzed using CytoHubba, and the interaction network was constructed by selecting the top 15 genes based on the MCC, as shown in Fig. 3D. The hub genes in the regulatory network were identified through a combination of MCC, DMNC, MNC, and Degree. This resulted in the identification of *Fdps, Lss, Sqle, Nsdhl,* and *Hmgcs2* as hub genes (Fig. 3E). Detailed information on the hub genes is provided in Table 2.

**Table 2:** The analysis results of hub genes

Gene	Full name	Log2FC	*P*-adjust
*Nsdhl*	NAD(P) dependent steroid dehydrogenase-like	1.508	2.43 × 10^− 89^
*Fdps*	farnesyl diphosphate synthetase	2.030	1.76 × 10^− 280^
*Hmgcs2*	3-hydroxy-3-methylglutaryl-Coenzyme A synthase 2	-2.487	3.57 × 10^− 73^
*Lss*	lanosterol synthase	1.702	6.29 × 10^− 114^
*Sqle*	squalene epoxidase	1.158	6.89 × 10^− 141^

### Validation of hub genes in GEO dataset

**Fig. 4:**
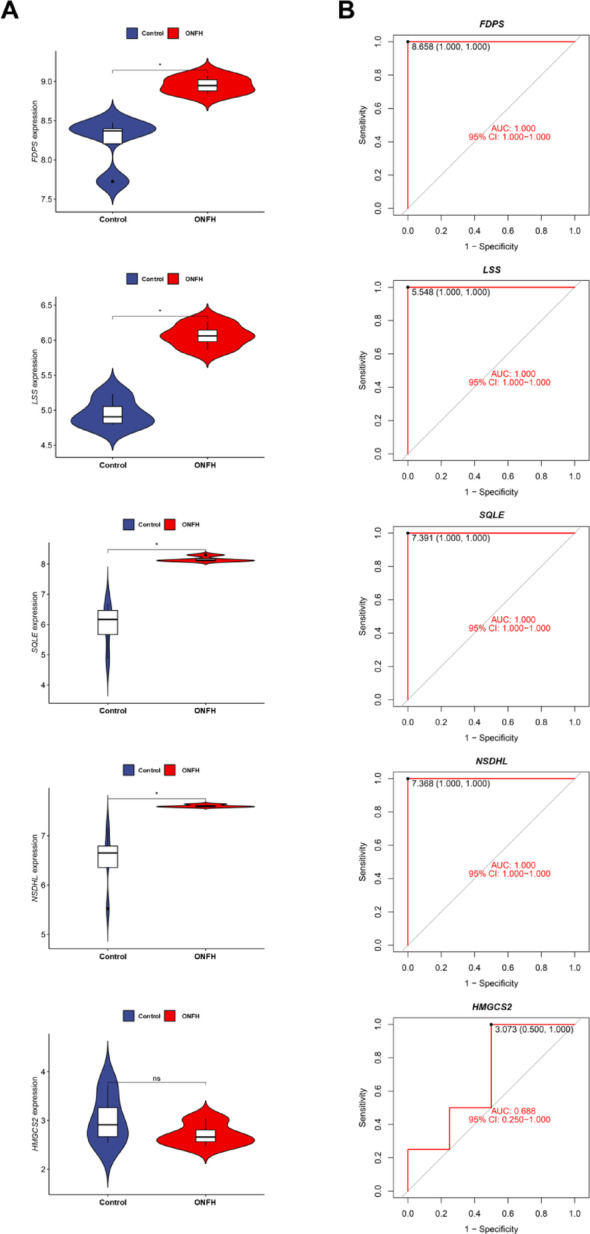
Validation of hub genes in GEO dataset. (**A**. Violin plot of 5 hub genes in GSE74089. **B**. ROC curve of 5 hub genes in GSE74089. *P < 0.05, ns means no significance)

The functionality of the hub genes was further validated by selecting the GSE74089 dataset from the GEO database for verification, which included data from 4 patients with ONFH and 4 healthy controls. The diagnostic value of the five hub genes was evaluated using the GSE74089 dataset. Violin plots of the five hub genes (*LSS, NSDHL, HMGCS2, SQLE, FDPS*) were constructed based on the GSE74089 dataset, as shown in Fig. 4A. *FDPS, LSS, SQLE*, and *NSDHL* were markedly up-regulated in the ONFH group compared with the control group (*P* < 0.05), while *HMGCS2* showed no significant difference (*P* > 0.05). For diagnostic value assessment, ROC curves were plotted (Fig. 4B). All five hub genes had AUC values above 0.5 in GSE74089. *FDPS, LSS, SQLE,* and *NSDHL* exhibited higher AUC values, supporting their potential as biomarkers. Although *HMGCS2* did not show a significant expression difference, its ROC curve still indicated some diagnostic capability, suggesting possible clinical utility under specific conditions or in combination with other genes.

### Gene set enrichment analysis (GSEA)

**Fig. 5:**
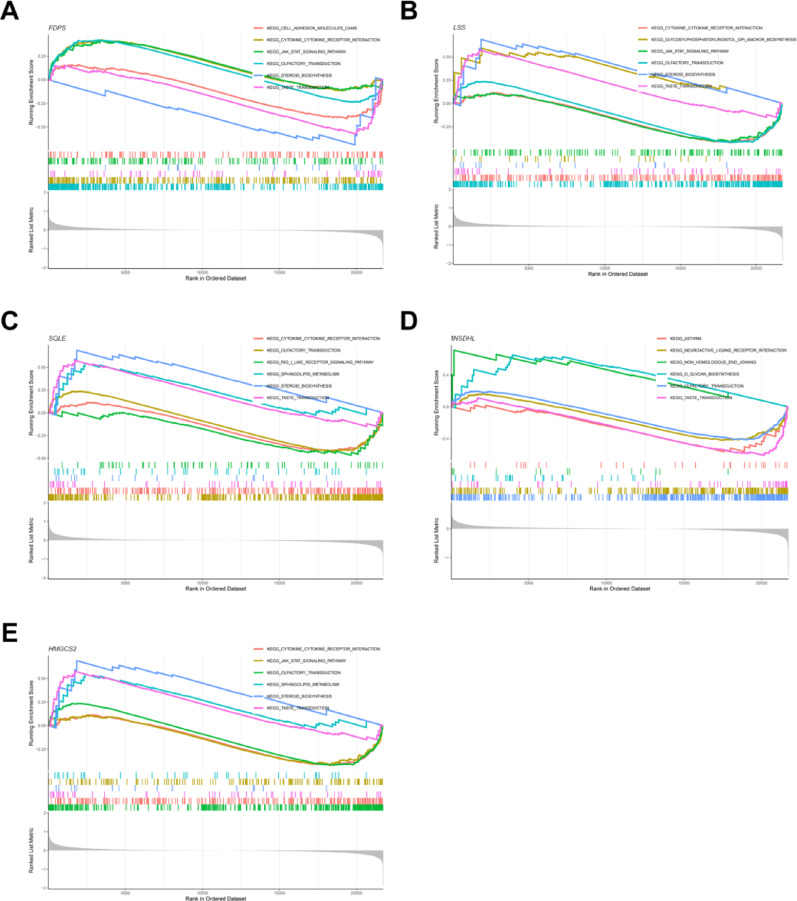
GSEA analysis. (**A**. *FDPS*. **B**. *LSS*. **C**. *SQLE*. **D**. *NSDHL*. **E**. *HMGCS2*.)

To investigate the potential role of hub genes in biological processes related to ONFH, we performed GSEA for five hub genes: *FDPS, LSS, SQLE, NSDHL,* and *HMGCS2*. As shown in Fig. 5A, *FDPS* is significantly enriched in pathways such as CYTOKINE RECEPTOR INTERACTION and JAK-STAT SIGNALING PATHWAY, while showing negative enrichment in pathways such as STEROID BIOSYNTHESIS and TASTE TRANSDUCTION. *LSS* is significantly enriched in pathways such as STEROID BIOSYNTHESIS and GLYCOSYLPHOSPHATIDYLINOSITOL (GPI) ANCHOR BIOSYNTHESIS, while showing negative enrichment in pathways such as CYTOKINE RECEPTOR INTERACTION and OLFACTORY TRANSDUCTION, as shown in Fig. 5B. *SQLE* is significantly enriched in pathways such as STEROID BIOSYNTHESIS and TASTE TRANSDUCTION, while showing negative enrichment in pathways such as RIG-I-LIKE RECEPTOR SIGNALING PATHWAY and CYTOKINE RECEPTOR INTERACTION, as shown in Fig. 5C. *NSDHL* is significantly enriched in pathways such as NON-HOMOLOGOUS END JOINING and GLYCAN BIOSYNTHESIS, while showing negative enrichment in pathways such as TASTE TRANSDUCTION and ASTHMA, as shown in Fig. 5D. *HMGCS2* is significantly enriched in pathways such as STEROID BIOSYNTHESIS and TASTE TRANSDUCTION, while showing negative enrichment in pathways such as CYTOKINE RECEPTOR INTERACTION and JAK-STAT SIGNALING PATHWAY, as shown in Fig. 5E.

### Interaction networks

**Fig. 6:**
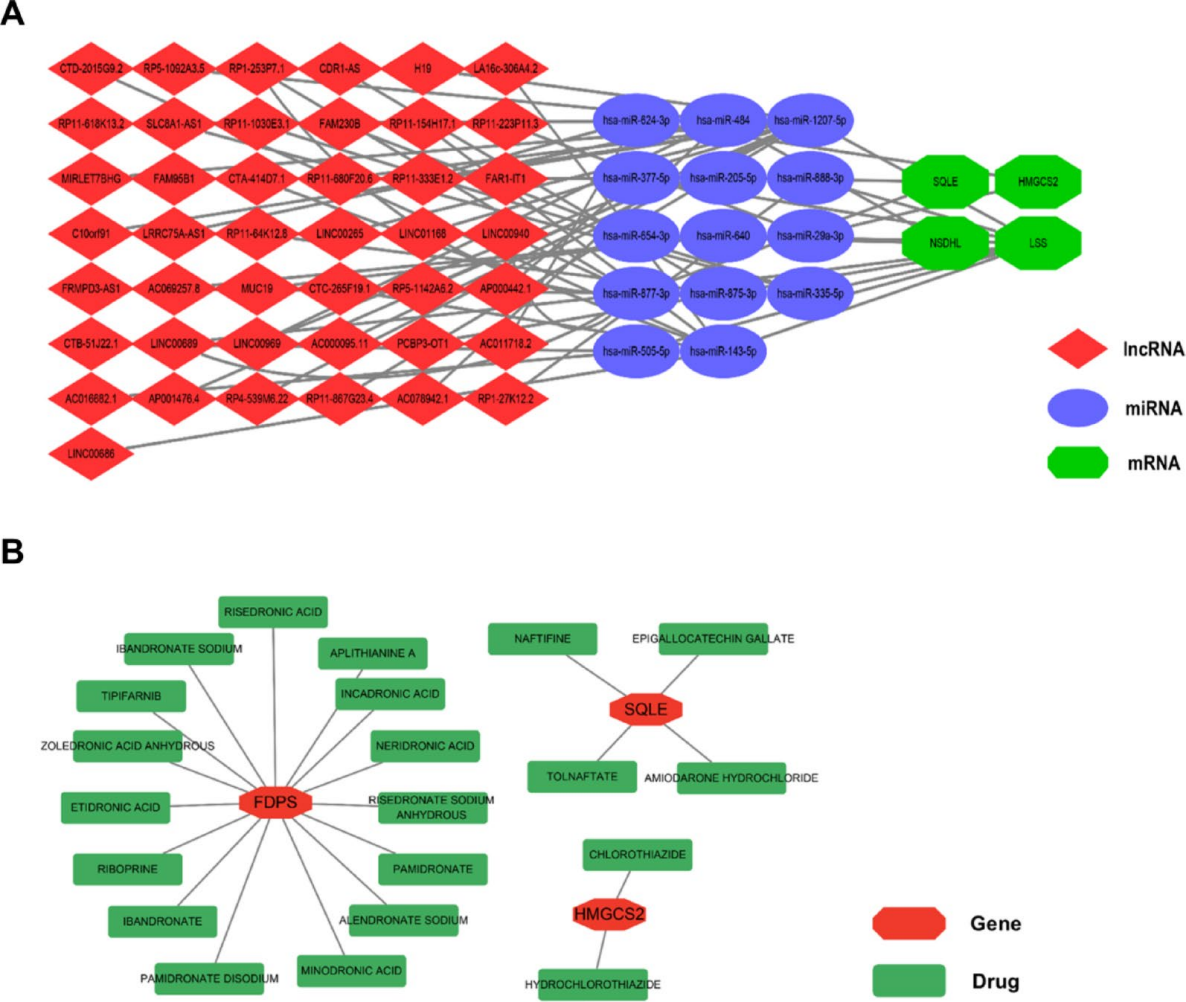
Interaction network. (**A**. The lncRNA-miRNA-mRNA network. **B**. The drug-hub gene interaction network)

Through prediction, a total of 43 upstream lncRNAs and 14 miRNAs related to each hub gene were identified. Based on these predicted results, the lncRNA-miRNA-mRNA regulatory network for ONFH was constructed, as shown in Fig. 6A and Table S3. A total of 21 drugs interacting with 5 hub genes were screened using DGIdb (Fig. 6B). Detailed information about the drugs and their corresponding targets is shown in Table S4. Among these, *FDPS* was the most frequently targeted gene by the majority of the drugs (15/21). The pairs with the highest interaction scores are: CHLOROTHIAZIDE—*HMGCS2* (interaction score: 7.457686); NAFTIFINE—*SQLE* (interaction score: 6.525475); and RIBOPRINE—*FDPS* (interaction score: 6.525475).

### Validation of hub genes through qRT-PCR

**Fig. 7:**
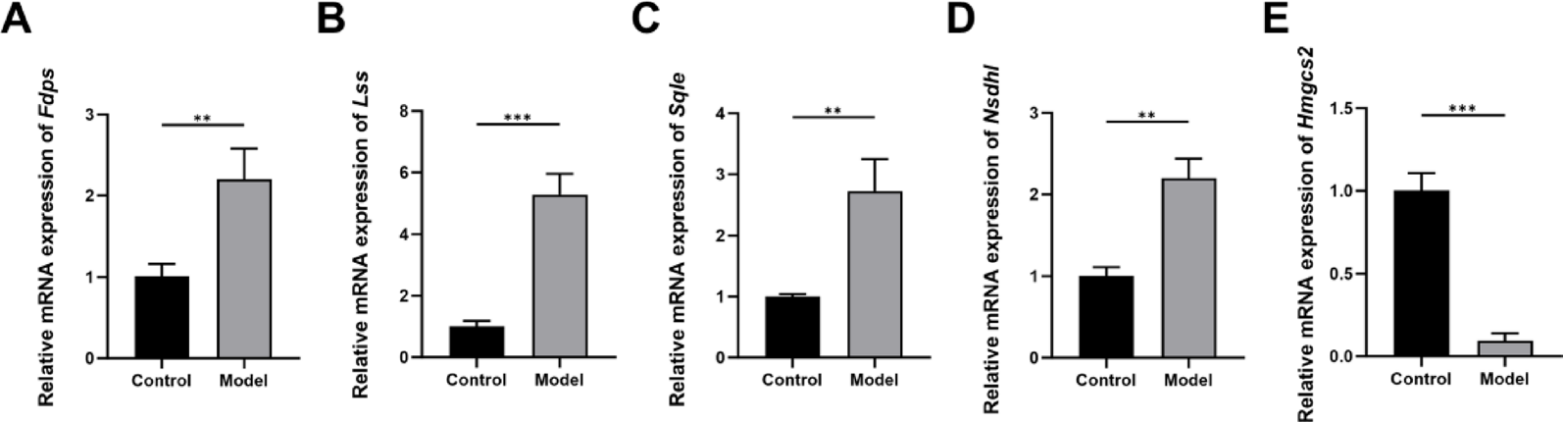
Validation of hub genes through qRT-PCR. (**A**. *Fdps*. **B**. *Lss*. **C**. *Sqle*. **D**. *Nsdhl*. **E**. *Hmgcs2*. n = 3. *P < 0.05, **P < 0.01, ***P < 0.001, ns means no significance)

In the ONFH cell model, the expression of core genes was verified by qRT-PCR to account for potential false positives in the sequencing data. The results are shown in Fig. 7. Compared to the control group, treatment with dexamethasone significantly increased the expression levels of *Fdps*, *Lss, Sqle*, and *Nsdhl* (*P* < 0.001), while significantly downregulating the expression of *Hmgcs2* (*P* < 0.001). The expression changes of these genes are consistent with the findings from high-throughput sequencing, thereby confirming the accuracy and reliability of the sequencing results.

### Effects of SQLE on the proliferation, apoptosis, and cell cycle of MC3T3-E1 induced by dexamethasone

**Fig. 8:**
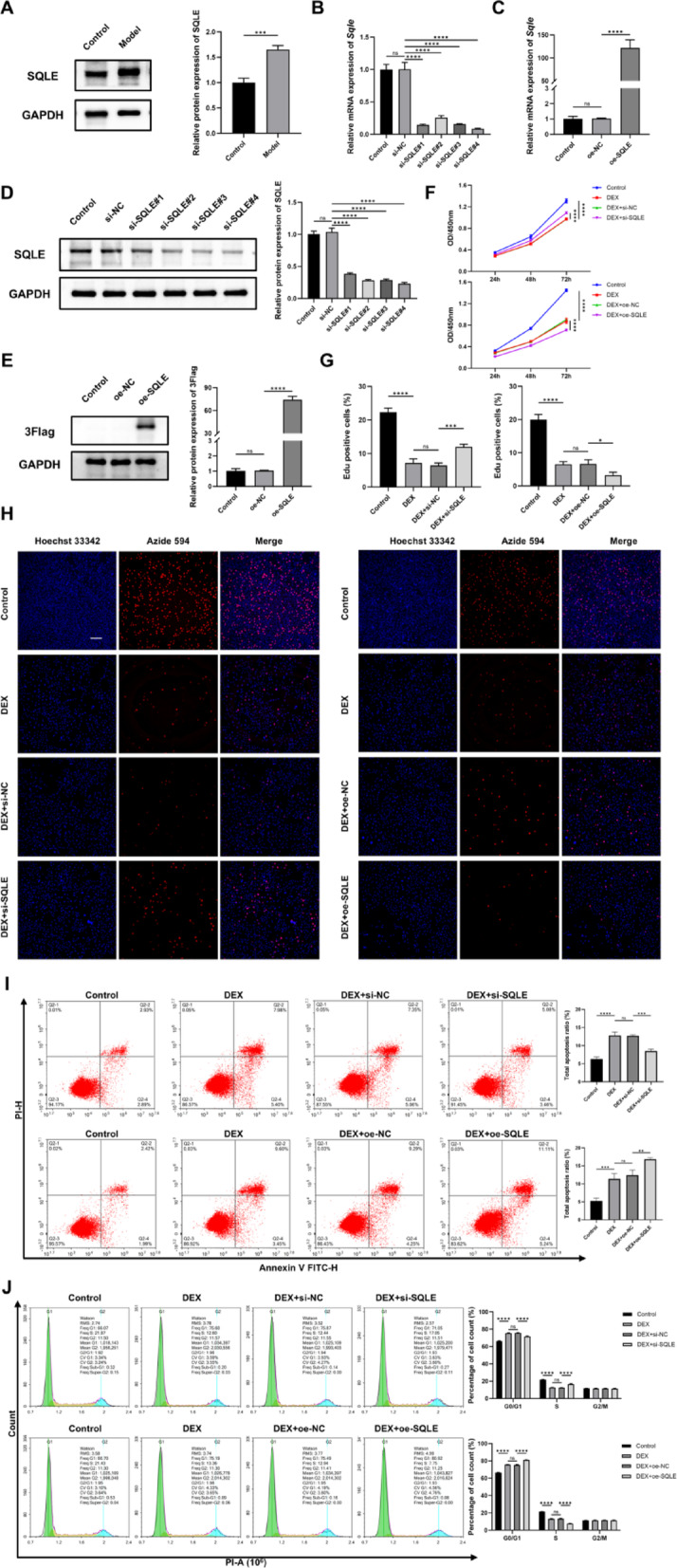
Effects of SQLE on the proliferation, apoptosis, and cell cycle of MC3T3-E1 induced by dexamethasone. (**A**. The protein expression of SQLE. **B**, **D**. The knockdown efficiency was detected by qRT-PCR and WB. **C**, **E**. The overexpression efficiency was detected by qRT-PCR and WB. F. CCK-8 detects cell viability. **G**, **H**. EdU assay. **I**. Flow cytometry detects cell apoptosis. **J**. Flow cytometry detects the cell cycle. n = 3. *P < 0.05, **P < 0.01, ***P < 0.001, ****P < 0.0001, ns means no significance. Scale bars = 200 μm)

The WB results showed that the expression of SQLE in the model group was also significantly increased (*P* < 0.001), as shown in Fig. 8A. Then, siRNA sequences and overexpression plasmids targeting SQLE were constructed. The knockdown and overexpression effects were subsequently validated by PCR and WB, as shown in Fig. 8B–E. si-SQLE#4 was selected for further experiments.

The effect of SQLE on cell viability was evaluated by CCK-8 and the EDU staining. The results showed that the inhibition of cell viability induced by dexamethasone could be significantly alleviated by knocking down SQLE, and the difference was statistically significant (*P* < 0.001). After overexpression of SQLE, cell viability was further inhibited (*P* < 0.05), as shown in Fig. 8F–H. Apoptosis and cell cycle were detected by flow cytometry. The results showed that, compared with the model group, the cell apoptosis rate decreased significantly after SQLE knockdown, indicating that SQLE knockdown can effectively inhibit apoptosis (*P* < 0.001). Conversely, the apoptosis rate in cells overexpressing SQLE was further elevated (*P* < 0.01), as shown in Fig. 8I.

Meanwhile, the results showed that dexamethasone treatment blocked the cell cycle in the G1 phase, and the proportion of S phase cells decreased. After knocking down SQLE, the proportion of G1 phase cells decreased, while the proportion of G2 phase cells increased, indicating that cell cycle arrest was alleviated (*P* < 0.0001). Conversely, in the cell group overexpressing SQLE, the proportion of G1 phase cells was further increased, and the proportion of S phase cells was decreased, leading to further blockage of the cell cycle in the G1 phase (*P* < 0.0001), as shown in Fig. 8J.

### Effect of SQLE on the osteogenic differentiation of MC3T3-E1 induced by dexamethasone

In this study, qRT-PCR and WB were used to detect the expression of osteogenic-related genes and proteins. Additionally, ALP staining was performed to evaluate osteogenic differentiation.

**Fig. 9:**
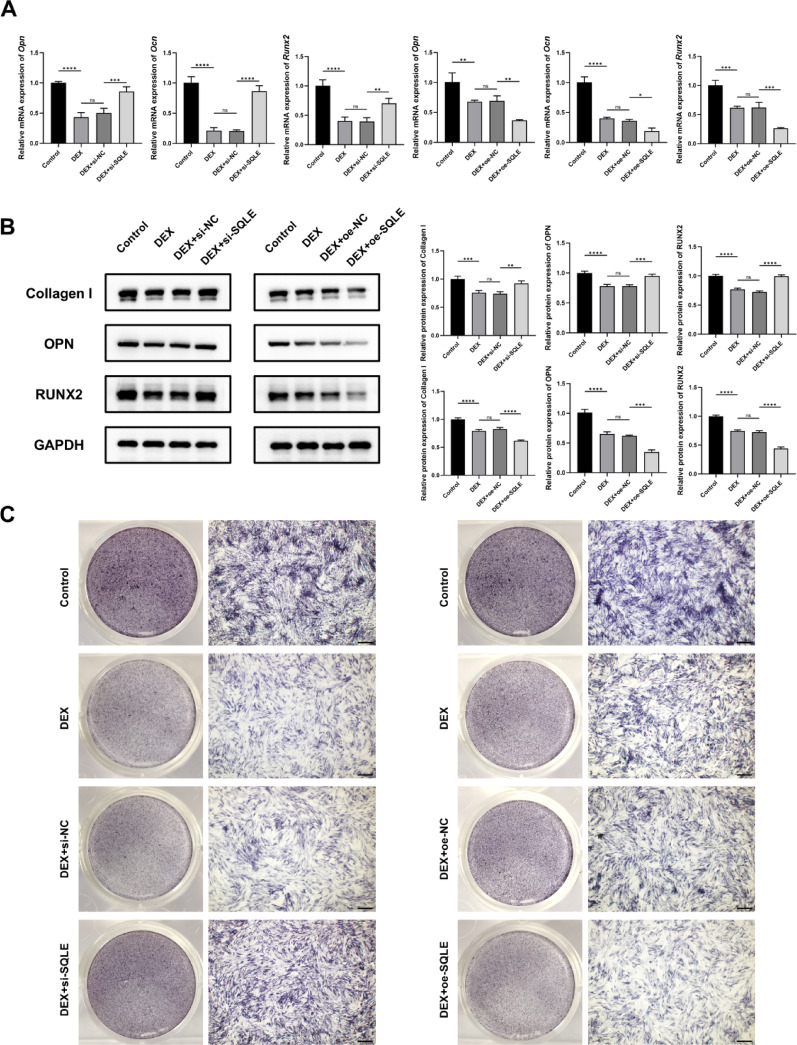
Effect of SQLE on the osteogenic differentiation of MC3T3-E1 induced by dexamethasone. (**A**. The mRNA expressions of *Opn, Ocn,* and *Runx2*. **B**. The protein expressions of Collagen I, RUNX2, and OPN. **C**. ALP staining. n = 3. *P < 0.05, **P < 0.01, ***P < 0.001, ****P < 0.0001, ns means no significance. Scale bars = 500 μm)

The qRT-PCR results showed that, as depicted in Fig. 9A, the expression levels of *Opn, Ocn*, and *Runx2* were significantly inhibited after dexamethasone treatment. However, after SQLE knockdown, the expression levels of these genes were significantly increased (*P* < 0.01). In contrast, overexpression of SQLE further suppressed the expression of *Opn, Ocn*, and *Runx2* (*P* < 0.05).

The WB results showed a similar trend in Fig. 9B. After dexamethasone treatment, the expressions of OPN, RUNX2, and Collagen I were significantly inhibited. SQLE knockdown significantly increased the expressions of OPN, RUNX2, and Collagen I (*P* < 0.01), while overexpression of SQLE further inhibited these expressions (*P* < 0.001).

The results of ALP staining are shown in Fig. 9C. The results showed that SQLE knockdown could reverse the inhibition of osteogenic differentiation induced by dexamethasone, with a significant increase in the ALP staining positive area. Conversely, after transfection with the overexpression plasmid, the osteogenic differentiation was further inhibited, and the positive area of ALP staining was reduced, indicating that SQLE overexpression inhibited osteogenic differentiation.

### Effect of SQLE on dexamethasone-induced MC3T3-E1 damage through mediating ferroptosis

**Fig. 10:**
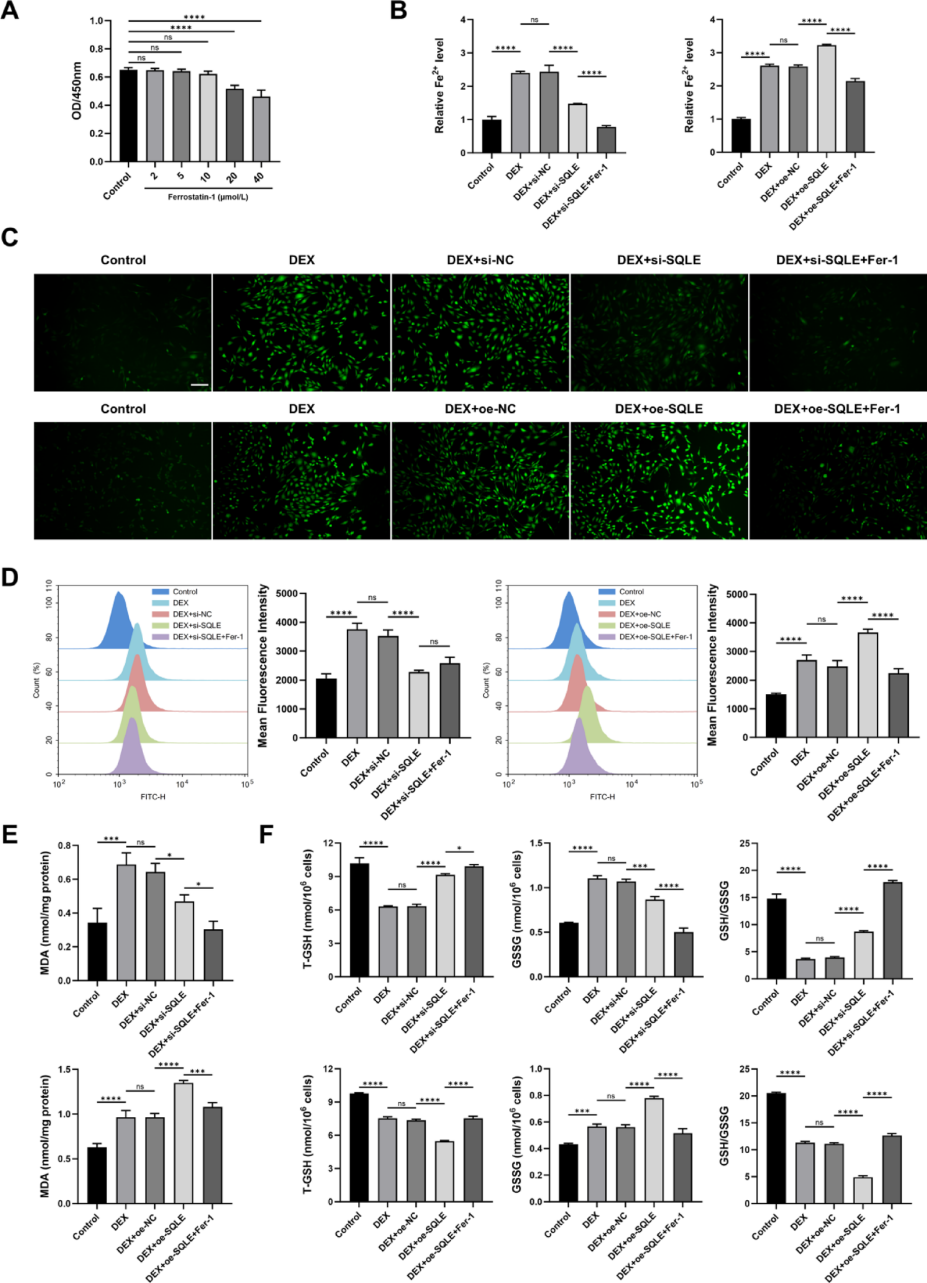
Effect of SQLE on dexamethasone-induced MC3T3-E1 damage through mediating ferroptosis. (**A**. CCK-8 detects cell viability. **B**. Intracellular Fe.^2^⁺ levels. **C**. Representative images of ROS. **D**. Flow cytometry analysis of ROS. **E**. The MDA levels. **F**. Intracellular of T-GSH, GSSG, and the GSH/GSSG ratio. n = 3. *P < 0.05, **P < 0.01, ***P < 0.001, ****P < 0.0001, ns means no significance. Scale bars = 200 μm)

To investigate the potential involvement of ferroptosis in SQLE-mediated osteogenic dysfunction, the optimal concentration of the ferroptosis inhibitor ferrostatin-1 (Fer-1) was determined by CCK-8 assay as shown in Fig. 10A. Subsequent experiments were conducted using a concentration of 10 μmol/L.

Further tests indicated that DEX significantly increased intracellular ferrous iron (Fe^2^⁺), malondialdehyde (MDA), and reactive oxygen species (ROS) levels, while reducing glutathione (GSH) content. SQLE knockdown attenuated these changes, whereas SQLE overexpression further exacerbated them. Importantly, co-treatment with Fer-1 reversed the effects of SQLE overexpression and enhanced the protective effects of SQLE knockdown (*P* < 0.05). As shown in Fig. 10B–F.

These results suggest that SQLE promotes ferroptosis under glucocorticoid exposure, thereby contributing to impaired osteogenesis, and that inhibiting ferroptosis can mitigate this process.

### Effect of the SQLE inhibitor terbinafine on glucocorticoid-induced ONFH in vivo

**Fig. 11:**
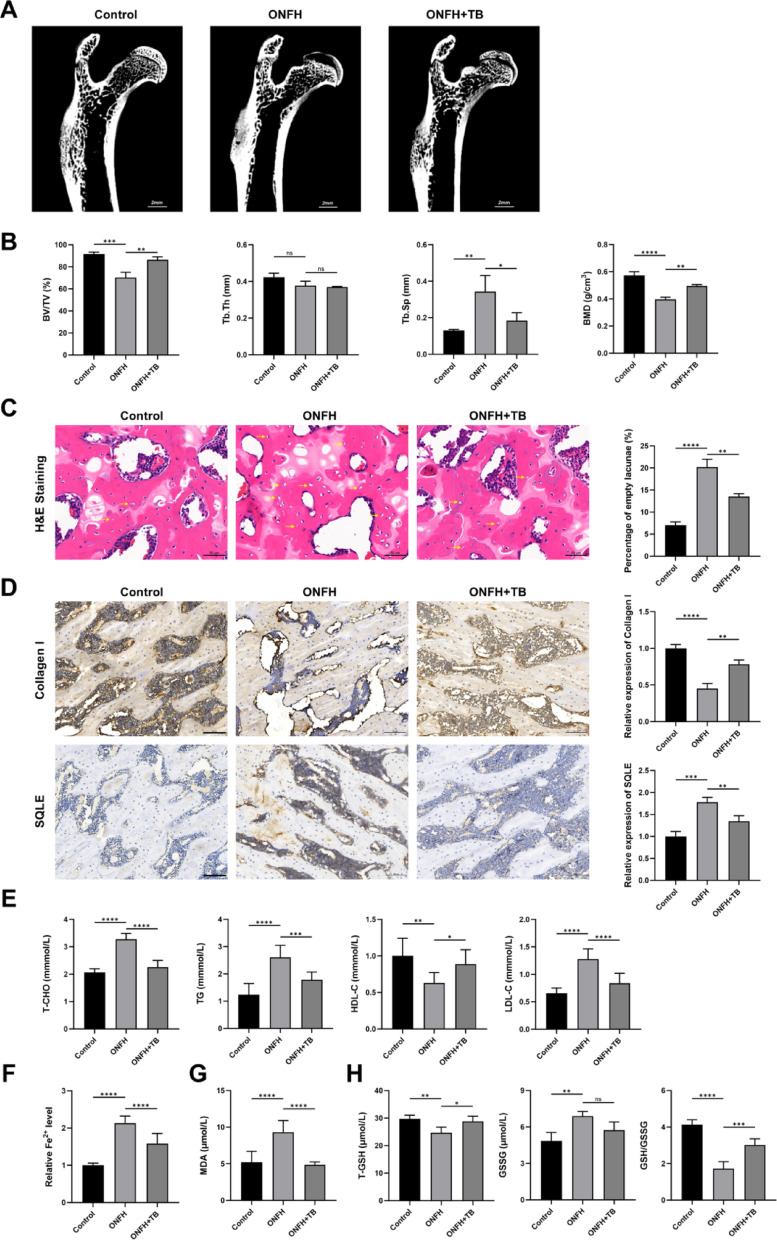
Effect of the SQLE inhibitor terbinafine on glucocorticoid-induced ONFH in vivo. (**A**. Micro-CT images. Scale bars = 2 mm. **B**. Quantitative analysis of BV/TV, Tb.Th, Tb.Sp, and BMD. **C**. H&E staining. Scale bars = 50 μm. **D**. Immunohistochemical staining. Scale bars = 100 μm. **E**. Serum T-CHO, TG, HDL-C, and LDL-C levels. **F**. Serum Fe.^2^⁺ levels. **G**. Serum MDA levels. **H**. Serum T-GSH, GSSG, and GSH/GSSG ratio. n = 6. *P < 0.05, **P < 0.01, ***P < 0.001, ****P < 0.0001, ns means no significance)

In this experiment, a rat model of glucocorticoid-induced ONFH was successfully established using combined lipopolysaccharide and methylprednisolone administration. Micro-CT analysis revealed a marked reduction in bone volume and trabecular integrity in the model group. H&E staining further confirmed the success of the model by showing typical features of osteonecrosis, including empty lacunae and trabecular disruption. In the treatment group receiving the SQLE inhibitor terbinafine, micro-CT and histological parameters were notably improved (*P* < 0.05), as shown in Fig. 11A–C.

As shown in Fig. 11D, immunohistochemical staining showed increased expression of SQLE and decreased expression of collagen I in the model group, while terbinafine treatment partially restored collagen I levels and reduced SQLE expression, suggesting a protective effect (*P* < 0.01).

Serum biochemical analysis revealed that the model group had significantly elevated levels of total cholesterol (T-CHO), triglycerides (TG), low-density lipoprotein cholesterol (LDL-C), and decreased high-density lipoprotein cholesterol (HDL-C). These lipid abnormalities were significantly ameliorated in the treatment group (*P* < 0.05) in Fig. 11E.

Additionally, serum levels of ferrous iron (Fe^2^⁺) and MDA were elevated, while GSH was reduced in the model group—changes consistent with ferroptosis activation. Treatment with terbinafine reversed these alterations, indicating that SQLE inhibition can mitigate lipid peroxidation and ferroptosis in vivo (*P* < 0.05) in Fig. 11F–H.

Together, these results demonstrate that targeting SQLE alleviates steroid-induced bone loss and ferroptosis, potentially by improving lipid metabolism and reducing oxidative stress.

## Discussion

In this study, we utilized transcriptome sequencing combined with bioinformatics analysis to investigate the differentially expressed genes in osteoblasts damaged by dexamethasone. The results demonstrated that dexamethasone treatment significantly altered the gene expression profile of osteoblasts, including 197 genes associated with lipid metabolism. These genes exhibited significant expression changes in ONFH and are closely linked to the lipid metabolism pathway, which may play a crucial role in the pathogenesis and progression of ONFH. To identify key regulatory genes, a PPI network was constructed, from which five hub genes (*Lss, Sqle, Hmgcs2, Fdps*, and *Nsdhl*) were identified. These genes may regulate cell proliferation and differentiation through the modulation of lipid metabolism, thereby contributing significantly to glucocorticoid-induced alterations in bone.

The 3-hydroxy-3-methylglutaryl-Coenzyme A synthase 2 (HMGCS2) serves as a pivotal enzyme in the pathway of ketone body synthesis, exerting regulatory control over both fatty acid oxidation and ketone body production processes [42]. The upregulation of HMGCS2 in tumor tissues is significantly associated with unfavorable clinical prognosis in patients, indicating its potential as a promising prognostic biomarker for tumors [43, 44]. Farnesyl diphosphate synthase (FDPS) is a pivotal enzyme in the mevalonate pathway, facilitating the enzymatic conversion of isopentenyl pyrophosphate and dimethylallyl pyrophosphate into geranyl pyrophosphate (GPP) and farnesyl pyrophosphate (FPP) [45]. Nitrogen-containing bisphosphonates (NBPs) demonstrate inhibitory effects on FPPS, thereby effectively impeding the osteogenic differentiation and calcification of vascular smooth muscle cells (VSMCs) [46]. Lanosterol synthase (LSS) is an enzyme that catalyzes the conversion of 2,3(S)-oxidosqualene into lanosterol, which is a crucial step in the biosynthesis process of cholesterol [47]. Knockdown of LSS can promote the formation of an immunosuppressive microenvironment by interacting with 2,3-oxidosqualene and binding to the PD-L1 protein, thereby accelerating malignant tumor progression [48]. The NAD(P)-dependent steroid dehydrogenase-like (NSDHL) gene encodes a sterol dehydrogenase or decarboxylase that plays a crucial role in cholesterol biosynthesis by catalyzing the conversion of cholesterol precursors into cholesterol [49]. NSDHL is highly expressed in breast cancer tissues, and its knockdown substantially inhibits the proliferation and migration of tumor cells [50].

Squalene epoxidase (SQLE), also known as squalene monooxygenase, acts as a crucial rate-limiting enzyme in the cholesterol biosynthetic pathway and facilitates the stereospecific conversion of squalene to 2,3(S)-oxidosqualene [51].

In patients with hepatocellular carcinoma (HCC) induced by non-alcoholic fatty liver disease (NAFLD), SQLE facilitates the proliferation of NAFLD-HCC cells by enhancing the biosynthesis of cholesterol esters [52]. SQLE also attenuates CD8 + T cell function and enhances the activity of myeloid-derived suppressor cells (MDSCs) through the accumulation of its metabolite cholesterol in the tumor microenvironment. This induces impaired anti-tumor responses in metabolic dysfunction-associated steatohepatitis-associated hepatocellular carcinoma (MASH-HCC) [53]. Polyphyllin I (PPI) directly binds to the SQLE protein, disrupting the SREBP-2/HMGCR/SQLE/LSS pathway and consequently interfering with cholesterol biosynthesis. This disruption leads to cholesterol accumulation and may contribute to hepatotoxicity [54]. In other diseases, SQLE mediates colorectal carcinogenesis through its endogenous effects and regulation of the gut microbiota-metabolite axis [55]. Silencing SQLE effectively suppresses the progression of osteosarcoma and enhances chemosensitivity by modulating cholesterol levels and inhibiting the FAK/PI3K/Akt/mTOR signaling pathway [56]. SQLE promotes tumor growth by alleviating endoplasmic reticulum stress and activating the lipid raft-regulated Src/PI3K/Akt signaling pathway in pancreatic cancer [41].

Although SQLE has been less studied in non-tumor diseases, given its crucial role in regulating cholesterol metabolism, it can be speculated that SQLE may play a role in the pathogenesis and therapeutic strategies of various diseases, such as ONFH. In this study, based on transcriptome sequencing, SQLE was identified as one of the hub genes and may play a significant role in glucocorticoid-induced osteoblast damage. In vitro experiments showed that SQLE knockdown reversed the dexamethasone-induced decrease in cell viability and the impairment of osteogenic differentiation in osteoblasts. Conversely, SQLE overexpression exacerbated the osteoblast dysfunction induced by dexamethasone.

SQLE may contribute to the development of ONFH through multiple pathways. Abnormal SQLE activity may lead to cholesterol metabolism disorders, and the accumulation of cholesterol metabolites can trigger local inflammatory responses and oxidative stress, eventually causing cell damage and necrosis [57, 58]. SQLE may also regulate several signaling pathways linked to the proliferation and differentiation of bone cells, such as the Wnt/β-catenin and PI3K/Akt pathways, thereby influencing the repair and regenerative capacity of bone tissue [41, 56]. Additionally, SQLE may influence the onset and progression of ONFH by modulating ferroptosis pathways.

Ferroptosis, also known as iron-dependent cell death, is a significant regulated form of cellular death characterized by the accumulation of lipid peroxides. Cholesterol oxidation and fatty acid metabolism are closely associated with ferroptosis [59]. SQLE may confer resistance to ferroptosis through the promotion of cholesterol synthesis, alteration of lipid composition, and regulation of ferroptosis-related signaling pathways [60–62].

Meanwhile, ferroptosis is also closely associated with the onset and progression of ONFH. Following glucocorticoid induction, bone cells and osteoblasts are damaged, accompanied by iron overload and increased oxidative stress [63]. In the serum and bone tissue of patients with non-traumatic ONFH, the expression of ferroptosis-related factors GPX4 and SLC7A11 is significantly decreased, suggesting their potential as biomarkers for ONFH progression [64]. Therefore, regulating the process of ferroptosis may help alleviate the progression of ONFH in patients [65–67]. Our current research findings also confirm that modulating SQLE expression can affect the ferroptosis phenotype in glucocorticoid-induced osteoblasts, thereby further supporting the association between SQLE and osteonecrosis of the femoral head.

At present, several inhibitors of SQLE, including terbinafine and naftifine, have been developed and applied clinically. Many studies have demonstrated that these drugs may exert therapeutic effects on related diseases through the regulation of lipid metabolism, alleviation of oxidative stress, and reduction of inflammatory responses [41, 53, 68, 69]. However, the application of SQLE inhibitors in the treatment of ONFH still faces some challenges, such as the specificity, safety of the drugs and their impact on osteocyte function.

In vivo, a rat model of glucocorticoid-induced ONFH was established by the combined administration of lipopolysaccharide and high-dose methylprednisolone. The results demonstrated that SQLE expression was upregulated in the bone tissues of the model group, accompanied by changes in oxidative stress and ferroptosis-related markers, including reduced GSH, elevated MDA, and increased levels of Fe^2^⁺. Following terbinafine treatment, the related indicators showed a certain degree of improvement, and the steroid-induced lipid metabolism disorder was also alleviated. These findings suggest that SQLE may play a role in the pathogenesis of ONFH by regulating ferroptosis, and that targeting SQLE or modulating ferroptosis could serve as a promising therapeutic strategy for ONFH.

However, this study still has some limitations. Firstly, transcriptome analysis was conducted on MC3T3-E1 cells and was complemented by in-depth analysis using publicly available ONFH datasets; thus, the translational relevance should be interpreted with caution. Additionally, the potential mechanisms through which SQLE regulates ferroptosis and lipid signaling in bone tissue warrant deeper investigation in future studies.

## Conclusions

In this study, transcriptome sequencing was used to investigate the changes in gene expression during glucocorticoid-induced osteoblast damage. We conducted a comprehensive analysis of lipid metabolism-related genes in osteonecrosis of the femoral head and identified hub genes, including FDPS, LSS, SQLE, NSDHL and HMGCS2. Overexpression of SQLE not only suppresses osteoblast function but also exacerbates osteonecrosis by promoting cholesterol metabolism disorders and inducing ferroptosis. SQLE knockdown or its inhibitors can effectively mitigate glucocorticoid-induced osteogenic dysfunction, indicating that SQLE may serve as a promising therapeutic target for femoral head necrosis. These findings provide a theoretical foundation for the development of effective treatment strategies for ONFH.

## Supplementary Information

Below is the link to the electronic supplementary material.

Supplementary file1 (DOCX 16 kb)

Supplementary file2 (DOCX 26 kb)

Supplementary file3 (DOCX 19 kb)

Supplementary file4 (DOCX 17 kb)

Supplementary file5 (PDF 199 kb)

## Data Availability

The datasets analysed during the current study are available in the Gene Expression Omnibus (GEO) repository: https://www.ncbi.nlm.nih.gov/geo/query/acc.cgi?%20acc=GSE74089. All data are available from the corresponding author on reasonable request.

## References

[1] Li L, Zhao S, Leng Z, Chen S, Shi Y, Shi L, et al. Pathological mechanisms and related markers of steroid-induced osteonecrosis of the femoral head. Ann Med. 2024;56(1):2416070. 39529511 10.1080/07853890.2024.2416070 PMC11559024

[2] Quaranta M, Miranda L, Oliva F, Aletto C, Maffulli N. Osteotomies for avascular necrosis of the femoral head. Br Med Bull. 2021;137(1):98–111. 33454780 10.1093/bmb/ldaa044

[3] Kubo T, Ueshima K, Saito M, Ishida M, Arai Y, Fujiwara H. Clinical and basic research on steroid-induced osteonecrosis of the femoral head in Japan. J Orthop Sci. 2016;21(4):407–13. 27062553 10.1016/j.jos.2016.03.008

[4] Tan B, Li W, Zeng P, Guo H, Huang Z, Fu F, et al. Epidemiological study based on China osteonecrosis of the femoral head database. Orthop Surg. 2021;13(1):153–60. 33347709 10.1111/os.12857 PMC7862166

[5] Liu F, Wang W, Yang L, Wang B, Wang J, Chai W, et al. An epidemiological study of etiology and clinical characteristics in patients with nontraumatic osteonecrosis of the femoral head. J Res Med Sci. 2017;22:15. 28458706 10.4103/1735-1995.200273 PMC5367210

[6] Cui Q, Jo WL, Koo KH, Cheng EY, Drescher W, Goodman SB, et al. ARCO consensus on the pathogenesis of non-traumatic osteonecrosis of the femoral head. J Korean Med Sci. 2021;36(10):e65. 33724736 10.3346/jkms.2021.36.e65 PMC7961868

[7] Petek D, Hannouche D, Suva D. Osteonecrosis of the femoral head: pathophysiology and current concepts of treatment. EFORT Open Rev. 2019;4(3):85–97. 30993010 10.1302/2058-5241.4.180036 PMC6440301

[8] Sodhi N, Acuna A, Etcheson J, Mohamed N, Davila I, Ehiorobo JO, et al. Management of osteonecrosis of the femoral head. Bone Joint J. 2020;102-b(7_Supple_B):122–8. 32600203 10.1302/0301-620X.102B7.BJJ-2019-1611.R1

[9] Migliorini F, Maffulli N, Eschweiler J, Tingart M, Baroncini A. Core decompression isolated or combined with bone marrow-derived cell therapies for femoral head osteonecrosis. Expert Opin Biol Ther. 2021;21(3):423–30. 33297783 10.1080/14712598.2021.1862790

[10] Sadile F, Bernasconi A, Russo S, Maffulli N. Core decompression versus other joint preserving treatments for osteonecrosis of the femoral head: a meta-analysis. Br Med Bull. 2016;118(1):33–49. 27298230 10.1093/bmb/ldw010 PMC5127418

[11] Zhao D, Zhang F, Wang B, Liu B, Li L, Kim SY, et al. Guidelines for clinical diagnosis and treatment of osteonecrosis of the femoral head in adults (2019 version). J Orthop Translat. 2020;21:100–10. 32309135 10.1016/j.jot.2019.12.004 PMC7152793

[12] Migliorini F, Maffulli N, Baroncini A, Eschweiler J, Tingart M, Betsch M. Prognostic factors in the management of osteonecrosis of the femoral head: a systematic review. Surgeon. 2023;21(2):85–98. 34991986 10.1016/j.surge.2021.12.004

[13] Wu T, Jiang Y, Tian H, Shi W, Wang Y, Li T. Systematic analysis of hip-preserving treatment for early osteonecrosis of the femoral head from the perspective of bibliometrics (2010–2023). J Orthop Surg Res. 2023;18(1):959. 38093378 10.1186/s13018-023-04435-8 PMC10717545

[14] Baek SH, Kim KH, Lee WK, Hong W, Won H, Kim SY. Abnormal lipid profiles in nontraumatic osteonecrosis of the femoral head: a comparison with osteoarthritis using propensity score matching. J Bone Joint Surg Am. 2022;104(Suppl 2):19–24. 35389903 10.2106/JBJS.20.00520

[15] Chang C, Greenspan A, Gershwin ME. The pathogenesis, diagnosis and clinical manifestations of steroid-induced osteonecrosis. J Autoimmun. 2020;110:102460. 32307211 10.1016/j.jaut.2020.102460

[16] Shao W, Wang P, Lv X, Wang B, Gong S, Feng Y. Unraveling the role of endothelial dysfunction in osteonecrosis of the femoral head: a pathway to new therapies. Biomedicines. 2024. 10.3390/biomedicines12030664. 38540277 10.3390/biomedicines12030664 PMC10967783

[17] Ma M, Tan Z, Li W, Zhang H, Liu Y, Yue C. Osteoimmunology and osteonecrosis of the femoral head. Bone Joint Res. 2022;11(1):26–8. 35045723 10.1302/2046-3758.111.BJR-2021-0467.R1 PMC8801166

[18] Wang Y, Ma X, Guo J, Li Y, Xiong Y. Correlation between ESR1 and APOE gene polymorphisms and risk of osteonecrosis of the femoral head: a case-control study. J Orthop Surg Res. 2023;18(1):968. 38102657 10.1186/s13018-023-04447-4 PMC10722694

[19] Jia D, Zhang Y, Li H, Guo C, Wu Y, Shi X, et al. Predicting steroid-induced osteonecrosis of the femoral head: role of lipid metabolism biomarkers and radiomics in young and middle-aged adults. J Orthop Surg Res. 2024;19(1):749. 39533346 10.1186/s13018-024-05245-2 PMC11558989

[20] Yu X, Zhang S, Zhang B, Dai M. Relationship of idiopathic femoral head necrosis with blood lipid metabolism and coagulation function: a propensity score-based analysis. Front Surg. 2022;9:938565. 36684312 10.3389/fsurg.2022.938565 PMC9852306

[21] Yu X, Dou S, Lu L, Wang M, Li Z, Wang D. Relationship between lipid metabolism, coagulation and other blood indices and etiology and staging of non-traumatic femoral head necrosis: a multivariate logistic regression-based analysis. J Orthop Surg Res. 2024;19(1):251. 38643101 10.1186/s13018-024-04715-x PMC11031896

[22] Chen Y, Tang B, Jiang W, Sun M, Zhang H, Tao Y, et al. Mir-486-5p attenuates steroid-induced adipogenesis and osteonecrosis of the femoral head via TBX2/P21 axis. Stem Cells. 2023;41(7):711–23. 37210668 10.1093/stmcls/sxad038

[23] Migliorini F, La Padula G, Oliva F, Torsiello E, Hildebrand F, Maffulli N. Operative management of avascular necrosis of the femoral head in skeletally immature patients: a systematic review. Life. 2022. 10.3390/life12020179. 35207467 10.3390/life12020179 PMC8879936

[24] Zheng QY, Tao Y, Geng L, Ren P, Ni M, Zhang GQ. Non-traumatic osteonecrosis of the femoral head induced by steroid and alcohol exposure is associated with intestinal flora alterations and metabolomic profiles. J Orthop Surg Res. 2024;19(1):236. 38609952 10.1186/s13018-024-04713-z PMC11015587

[25] Fang L, Zhang G, Wu Y, Li Z, Gao S, Zhou L. SIRT6 prevents glucocorticoid-induced osteonecrosis of the femoral head in rats. Oxid Med Cell Longev. 2022;2022:6360133. 36275897 10.1155/2022/6360133 PMC9584736

[26] Chen S, Zhou Y, Chen Y, Gu J. Fastp: an ultra-fast all-in-one FASTQ preprocessor. Bioinformatics. 2018;34(17):i884–90. 30423086 10.1093/bioinformatics/bty560 PMC6129281

[27] Kim D, Langmead B, Salzberg SL. HISAT: a fast spliced aligner with low memory requirements. Nat Methods. 2015;12(4):357–60. 25751142 10.1038/nmeth.3317 PMC4655817

[28] Liao Y, Smyth GK, Shi W. Featurecounts: an efficient general purpose program for assigning sequence reads to genomic features. Bioinformatics. 2014;30(7):923–30. 24227677 10.1093/bioinformatics/btt656

[29] Mcdermaid A, Monier B, Zhao J, Liu B, Ma Q. Interpretation of differential gene expression results of RNA-seq data: review and integration. Brief Bioinform. 2019;20(6):2044–54. 30099484 10.1093/bib/bby067 PMC6954399

[30] Han C, Shi C, Liu L, Han J, Yang Q, Wang Y, et al. Majorbio cloud 2024: update single-cell and multiomics workflows. Imeta. 2024;3(4):e217. 39135689 10.1002/imt2.217 PMC11316920

[31] Stelzer G, Rosen N, Plaschkes I, Zimmerman S, Twik M, Fishilevich S, et al. The GeneCards suite: from gene data mining to disease genome sequence analyses. Curr Protoc Bioinf. 2016;54(1):1–30. 10.1002/cpbi.5 27322403

[32] Szklarczyk D, Kirsch R, Koutrouli M, Nastou K, Mehryary F, Hachilif R, et al. The STRING database in 2023: protein-protein association networks and functional enrichment analyses for any sequenced genome of interest. Nucleic Acids Res. 2023;51(D1):D638-d646. 36370105 10.1093/nar/gkac1000 PMC9825434

[33] Doncheva NT, Morris JH, Gorodkin J, Jensen LJ. Cytoscape StringApp: network analysis and visualization of proteomics data. J Proteome Res. 2019;18(2):623–32. 30450911 10.1021/acs.jproteome.8b00702 PMC6800166

[34] Barrett T, Wilhite SE, Ledoux P, Evangelista C, Kim IF, Tomashevsky M, et al. Ncbi geo: archive for functional genomics data sets–update. Nucleic Acids Res. 2013;41(Database issue):D991-995. 23193258 10.1093/nar/gks1193 PMC3531084

[35] Ritchie ME, Phipson B, Wu D, Hu Y, Law CW, Shi W, et al. Limma powers differential expression analyses for RNA-sequencing and microarray studies. Nucleic Acids Res. 2015;43(7):e47. 25605792 10.1093/nar/gkv007 PMC4402510

[36] Cannon M, Stevenson J, Stahl K, Basu R, Coffman A, Kiwala S, et al. DGIdb 5.0: rebuilding the drug-gene interaction database for precision medicine and drug discovery platforms. Nucleic Acids Res. 2024;52(D1):D1227–35. 37953380 10.1093/nar/gkad1040 PMC10767982

[37] Shen Y, Zhang Y, Wang Q, Jiang B, Jiang X, Luo B. Microrna-877-5p promotes osteoblast differentiation by targeting EIF4G2 expression. J Orthop Surg Res. 2024;19(1):134. 38342889 10.1186/s13018-023-04396-y PMC10860299

[38] Chen Y, Wang X. miRDB: an online database for prediction of functional microRNA targets. Nucleic Acids Res. 2020;48(D1):D127–31. 31504780 10.1093/nar/gkz757 PMC6943051

[39] Mcgeary SE, Lin KS, Shi CY, Pham TM, Bisaria N, Kelley GM, et al. The biochemical basis of microRNA targeting efficacy. Science. 2019. 10.1126/science.aav1741. 31806698 10.1126/science.aav1741 PMC7051167

[40] Zhao X, Alqwbani M, Luo Y, Chen C, A G, Wei Y, Li D, Wang Q, Tian M, Kang P,. Glucocorticoids decreased Cx43 expression in osteonecrosis of femoral head: the effect on proliferation and osteogenic differentiation of rat BMSCs. J Cell Mol Med. 2021;25(1):484–98. 33205619 10.1111/jcmm.16103 PMC7810924

[41] Xu R, Song J, Ruze R, Chen Y, Yin X, Wang C, et al. Sqle promotes pancreatic cancer growth by attenuating ER stress and activating lipid rafts-regulated Src/PI3K/Akt signaling pathway. Cell Death Dis. 2023;14(8):497. 37542052 10.1038/s41419-023-05987-7 PMC10403582

[42] Xu Y, Ye X, Zhou Y, Cao X, Peng S, Peng Y, et al. Sodium butyrate activates HMGCS2 to promote ketone body production through SIRT5-mediated desuccinylation. Front Med. 2023;17(2):339–51. 36602721 10.1007/s11684-022-0943-0

[43] Wang J, Shidfar A, Ivancic D, Ranjan M, Liu L, Choi MR, et al. Overexpression of lipid metabolism genes and PBX1 in the contralateral breasts of women with estrogen receptor-negative breast cancer. Int J Cancer. 2017;140(11):2484–97. 28263391 10.1002/ijc.30680

[44] Wan S, Xi M, Zhao HB, Hua W, Liu YL, Zhou YL, et al. HMGCS2 functions as a tumor suppressor and has a prognostic impact in prostate cancer. Pathol Res Pract. 2019;215(8):152464. 31176575 10.1016/j.prp.2019.152464

[45] Jin T, Lu J, Lv Q, Gong Y, Feng Z, Ying H, et al. Farnesyl diphosphate synthase regulated endothelial proliferation and autophagy during rat pulmonary arterial hypertension induced by monocrotaline. Mol Med. 2022;28(1):94. 35962329 10.1186/s10020-022-00511-7 PMC9373289

[46] Xu W, Gong L, Tang W, Lu G. Nitrogen-containing bisphosphonate induces enhancement of OPG expression and inhibition of RANKL expression via inhibition of farnesyl pyrophosphate synthase to inhibit the osteogenic differentiation and calcification in vascular smooth muscle cells. BMC Cardiovasc Disord. 2024;24(1):494. 39289624 10.1186/s12872-024-04048-x PMC11406803

[47] Sun X, Zhang J, Liu H, Li M, Liu L, Yang Z, et al. Lanosterol synthase loss of function decreases the malignant phenotypes of HepG2 cells by deactivating the Src/MAPK signaling pathway. Oncol Lett. 2023;26(1):295. 37274468 10.3892/ol.2023.13881 PMC10236266

[48] Gao Y, Zhao K, Huang Y, Zhang D, Luo N, Peng X, et al. Lanosterol synthase deficiency promotes tumor progression by orchestrating PDL1-dependent tumor immunosuppressive microenvironment. MedComm. 2024;5(4):e528. 38606362 10.1002/mco2.528 PMC11006713

[49] Yoon SH, Kim HS, Kim RN, Jung SY, Hong BS, Kang EJ, et al. NAD(P)-dependent steroid dehydrogenase-like is involved in breast cancer cell growth and metastasis. BMC Cancer. 2020;20(1):375. 32366230 10.1186/s12885-020-06840-2 PMC7197182

[50] Chen M, Zhao Y, Yang X, Zhao Y, Liu Q, Liu Y, et al. Nsdhl promotes triple-negative breast cancer metastasis through the TGFβ signaling pathway and cholesterol biosynthesis. Breast Cancer Res Treat. 2021;187(2):349–62. 33864166 10.1007/s10549-021-06213-8

[51] Padyana AK, Gross S, Jin L, Cianchetta G, Narayanaswamy R, Wang F, et al. Structure and inhibition mechanism of the catalytic domain of human squalene epoxidase. Nat Commun. 2019;10(1):97. 30626872 10.1038/s41467-018-07928-x PMC6327030

[52] Liu D, Wong CC, Fu L, Chen H, Zhao L, Li C, et al. Squalene epoxidase drives NAFLD-induced hepatocellular carcinoma and is a pharmaceutical target. Sci Transl Med. 2018. 10.1126/scitranslmed.aap9840. 29669855 10.1126/scitranslmed.aap9840

[53] Wen J, Zhang X, Wong CC, Zhang Y, Pan Y, Zhou Y, et al. Targeting squalene epoxidase restores anti-PD-1 efficacy in metabolic dysfunction-associated steatohepatitis-induced hepatocellular carcinoma. Gut. 2024;73(12):2023–36. 38744443 10.1136/gutjnl-2023-331117 PMC11671884

[54] Li Z, Fan Q, Chen M, Dong Y, Li F, Wang M, et al. The interaction between polyphyllin I and SQLE protein induces hepatotoxicity through SREBP-2/HMGCR/SQLE/LSS pathway. J Pharm Anal. 2023;13(1):39–54. 36820075 10.1016/j.jpha.2022.11.005 PMC9937801

[55] Li C, Wang Y, Liu D, Wong CC, Coker OO, Zhang X, et al. Squalene epoxidase drives cancer cell proliferation and promotes gut dysbiosis to accelerate colorectal carcinogenesis. Gut. 2022;71(11):2253–65. 35232776 10.1136/gutjnl-2021-325851 PMC9554078

[56] Wang Y, Ma X, Xu E, Huang Z, Yang C, Zhu K, et al. Identifying squalene epoxidase as a metabolic vulnerability in high-risk osteosarcoma using an artificial intelligence-derived prognostic index. Clin Transl Med. 2024;14(2):e1586. 38372422 10.1002/ctm2.1586 PMC10875711

[57] Zhang J, Xu H, He Y, Zheng X, Lin T, Yang L, et al. Inhibition of KDM4A restricts SQLE transcription and induces oxidative stress imbalance to suppress bladder cancer. Redox Biol. 2024;77:103407. 39461328 10.1016/j.redox.2024.103407 PMC11543538

[58] Li G, Chen L, Bai H, Zhang L, Wang J, Li W. Depletion of squalene epoxidase in synergy with glutathione peroxidase 4 inhibitor RSL3 overcomes oxidative stress resistance in lung squamous cell carcinoma. Precis Clin Med. 2024;7(2):pbae011. 38779359 10.1093/pcmedi/pbae011 PMC11109822

[59] Sun Q, Liu D, Cui W, Cheng H, Huang L, Zhang R, et al. Cholesterol mediated ferroptosis suppression reveals essential roles of coenzyme Q and squalene. Commun Biol. 2023;6(1):1108. 37914914 10.1038/s42003-023-05477-8 PMC10620397

[60] Zhang R, Zhang L, Fan S, Wang L, Wang B, Wang L. Squalene monooxygenase (SQLE) protects ovarian cancer cells from ferroptosis. Sci Rep. 2024;14(1):22646. 39349544 10.1038/s41598-024-72506-9 PMC11442994

[61] Zhang L, Cao Z, Hong Y, He H, Chen L, Yu Z, et al. Squalene epoxidase: its regulations and links with cancers. Int J Mol Sci. 2024. 10.3390/ijms25073874. 38612682 10.3390/ijms25073874 PMC11011400

[62] Mao X, Wang L, Chen Z, Huang H, Chen J, Su J, et al. Scd1 promotes the stemness of gastric cancer stem cells by inhibiting ferroptosis through the SQLE/cholesterol/mTOR signalling pathway. Int J Biol Macromol. 2024;275(Pt 2):133698. 38972654 10.1016/j.ijbiomac.2024.133698

[63] Sun F, Zhou JL, Liu ZL, Jiang ZW, Peng H. Dexamethasone induces ferroptosis via P53/SLC7A11/GPX4 pathway in glucocorticoid-induced osteonecrosis of the femoral head. Biochem Biophys Res Commun. 2022;602:149–55. 35276555 10.1016/j.bbrc.2022.02.112

[64] Ye QH, Zhang P, Zhao YH, Zhu WX, Zhu HX, Wei BF. Decreased serum and local GPX4 and SLC7A11 expression correlates with disease severity in non-traumatic osteonecrosis of the femoral head. J Orthop Surg Res. 2025;20(1):477. 40380264 10.1186/s13018-025-05912-y PMC12084951

[65] Zuo R, Cao B, Kong L, Wang F, Li S, Shan H, et al. MiR-370-3p regulate TLR4/SLC7A11/GPX4 to alleviate the progression of glucocorticoids-induced osteonecrosis of the femoral head by promoting osteogenesis and suppressing ferroptosis. J Orthop Transl. 2025;51:337–58. 10.1016/j.jot.2024.10.014 PMC12206324 40584015

[66] Li W, Li W, Zhang W, Wang H, Yu L, Yang P, et al. Exogenous melatonin ameliorates steroid-induced osteonecrosis of the femoral head by modulating ferroptosis through GDF15-mediated signaling. Stem Cell Res Ther. 2023;14(1):171. 37400902 10.1186/s13287-023-03371-y PMC10318673

[67] Fan Y, Chen Z, Wang H, Jiang M, Lu H, Wei Y, et al. Isovitexin targets SIRT3 to prevent steroid-induced osteonecrosis of the femoral head by modulating mitophagy-mediated ferroptosis. Bone Res. 2025;13(1):18. 39865068 10.1038/s41413-024-00390-0 PMC11770138

[68] Chen M, Yang Y, Chen S, He Z, Du L. Targeting squalene epoxidase in the treatment of metabolic-related diseases: current research and future directions. PeerJ. 2024;12:e18522. 39588004 10.7717/peerj.18522 PMC11587872

[69] He L, Li H, Pan C, Hua Y, Peng J, Zhou Z, et al. Squalene epoxidase promotes colorectal cancer cell proliferation through accumulating calcitriol and activating CYP24A1-mediated MAPK signaling. Cancer Commun. 2021;41(8):726–46. 10.1002/cac2.12187 PMC8360641 34268906

