# Supplementary material for: Identification and validation of SQLE in steroid-induced osteonecrosis of the femoral head: a bioinformatics and experimental study
Source: J Orthop Surg Res. 2025 Oct 17;20:894. doi: 10.1186/s13018-025-06305-x (PMC12533337; doi:10.1186/s13018-025-06305-x)
Supplement: Supplementary file 1 — Supplementary file1 (DOCX 16 kb) [file 13018_2025_6305_MOESM1_ESM.docx]

**Table S1. The information of the siRNAs.**

| **Name** | **Sense (5ʹ to 3ʹ)** | **Antisense (5ʹ to 3ʹ)** |
| --- | --- | --- |
| si-NC (Negative control) | UUCUCCGAACGUGUCACGUTT | ACGUGACACGUUCGGAGAATT |
| si-SQLE#1 | GUCAUCGAGAGAGAUUUAATT | UUAAAUCUCUCUCGAUGACTT |
| si-SQLE#2 | GCCGAUUCAUCAUGAGUCUTT | AGACUCAUGAUGAAUCGGCTT |
| si-SQLE#3 | GGACUCUUCUCCAAGUUCATT | UGAACUUGGAGAAGAGUCCTT |
| si-SQLE#4 | GGGUUGCUUUCAAUAUUGUTT | ACAAUAUUGAAAGCAACCCTT |
