# Supplementary material for: Identification and validation of SQLE in steroid-induced osteonecrosis of the femoral head: a bioinformatics and experimental study
Source: J Orthop Surg Res. 2025 Oct 17;20:894. doi: 10.1186/s13018-025-06305-x (PMC12533337; doi:10.1186/s13018-025-06305-x)
Supplement: Supplementary file 2 — Supplementary file2 (DOCX 26 kb) [file 13018_2025_6305_MOESM2_ESM.docx]

**Table S2. GO and KEGG pathway enrichment analysis of DEGs (Top 10 in each term of GO).**

|  | **Category** | **Term** | **Description** | **Count** | **Padjust** |
| --- | --- | --- | --- | --- | --- |
| Up-regulated DEGs | BP | GO:0016126 | sterol biosynthetic process | 15 | 8.59E-19 |
|  |  | GO:0016125 | sterol metabolic process | 17 | 4.30E-15 |
|  |  | GO:0006695 | cholesterol biosynthetic process | 12 | 2.27E-14 |
|  |  | GO:1902653 | secondary alcohol biosynthetic process | 12 | 2.33E-14 |
|  |  | GO:0008203 | cholesterol metabolic process | 15 | 2.86E-13 |
|  |  | GO:0006694 | steroid biosynthetic process | 15 | 3.51E-13 |
|  |  | GO:1902652 | secondary alcohol metabolic process | 15 | 9.04E-13 |
|  |  | GO:1901617 | organic hydroxy compound biosynthetic process | 17 | 2.50E-12 |
|  |  | GO:0044281 | small molecule metabolic process | 49 | 1.48E-11 |
|  |  | GO:0046165 | alcohol biosynthetic process | 14 | 6.23E-11 |
|  | CC | GO:0005783 | endoplasmic reticulum | 29 | 1.39E-06 |
|  |  | GO:0043231 | intracellular membrane-bounded organelle | 111 | 1.39E-06 |
|  |  | GO:0043227 | membrane-bounded organelle | 113 | 1.39E-06 |
|  |  | GO:0005789 | endoplasmic reticulum membrane | 22 | 8.36E-06 |
|  |  | GO:0110165 | cellular anatomical entity | 204 | 1.11E-05 |
|  |  | GO:0005739 | mitochondrion | 32 | 5.60E-05 |
|  |  | GO:0031090 | organelle membrane | 40 | 0.003007 |
|  |  | GO:0043229 | intracellular organelle | 124 | 0.004118 |
|  |  | GO:0043226 | organelle | 126 | 0.004118 |
|  |  | GO:0005737 | cytoplasm | 64 | 0.004541 |
|  | MF | GO:0005515 | protein binding | 100 | 4.65E-08 |
|  |  | GO:0046983 | protein dimerization activity | 32 | 4.50E-06 |
|  |  | GO:0004812 | aminoacyl-tRNA ligase activity | 7 | 7.20E-05 |
|  |  | GO:0016875 | ligase activity, forming carbon-oxygen bonds | 7 | 7.20E-05 |
|  |  | GO:0016491 | oxidoreductase activity | 28 | 0.000113 |
|  |  | GO:0005488 | binding | 160 | 0.000164 |
|  |  | GO:0042803 | protein homodimerization activity | 21 | 0.000178 |
|  |  | GO:0042802 | identical protein binding | 34 | 0.000378 |
|  |  | GO:0003824 | catalytic activity | 84 | 0.000488 |
|  |  | GO:0016874 | ligase activity | 10 | 0.001415 |
|  | KEGG | mmu00100 | Steroid biosynthesis | 10 | 4.19E-13 |
|  |  | mmu04216 | Ferroptosis | 7 | 1.74E-05 |
|  |  | mmu00970 | Aminoacyl-tRNA biosynthesis | 7 | 2.30E-05 |
|  |  | mmu04978 | Mineral absorption | 7 | 6.42E-05 |
|  |  | mmu00900 | Terpenoid backbone biosynthesis | 5 | 0.000176 |
|  |  | mmu00250 | Alanine, aspartate and glutamate metabolism | 4 | 0.02218 |
|  |  | mmu00670 | One carbon pool by folate | 3 | 0.02831 |
|  |  | mmu00220 | Arginine biosynthesis | 3 | 0.02892 |
|  |  | mmu00270 | Cysteine and methionine metabolism | 4 | 0.055643 |
|  |  | mmu01040 | Biosynthesis of unsaturated fatty acids | 3 | 0.109097 |
| Down-regulated DEGs | BP | GO:0032502 | developmental process | 112 | 5.99E-19 |
|  |  | GO:0048856 | anatomical structure development | 82 | 1.31E-15 |
|  |  | GO:0030198 | extracellular matrix organization | 22 | 1.85E-15 |
|  |  | GO:0045229 | external encapsulating structure organization | 22 | 1.85E-15 |
|  |  | GO:0043062 | extracellular structure organization | 22 | 1.85E-15 |
|  |  | GO:0051239 | regulation of multicellular organismal process | 70 | 1.72E-13 |
|  |  | GO:2000026 | regulation of multicellular organismal development | 43 | 1.74E-12 |
|  |  | GO:0065007 | biological regulation | 179 | 2.14E-11 |
|  |  | GO:0007155 | cell adhesion | 34 | 2.30E-11 |
|  |  | GO:0050789 | regulation of biological process | 174 | 4.70E-11 |
|  | CC | GO:0031012 | extracellular matrix | 39 | 6.13E-26 |
|  |  | GO:0030312 | external encapsulating structure | 39 | 6.13E-26 |
|  |  | GO:0062023 | collagen-containing extracellular matrix | 32 | 2.19E-22 |
|  |  | GO:0005615 | extracellular space | 63 | 4.30E-20 |
|  |  | GO:0005576 | extracellular region | 32 | 6.43E-09 |
|  |  | GO:0042995 | cell projection | 40 | 3.93E-06 |
|  |  | GO:0043005 | neuron projection | 29 | 2.04E-05 |
|  |  | GO:0120025 | plasma membrane bounded cell projection | 37 | 2.04E-05 |
|  |  | GO:0009986 | cell surface | 21 | 2.08E-05 |
|  |  | GO:0005581 | collagen trimer | 8 | 3.91E-05 |
|  | MF | GO:0005102 | signaling receptor binding | 52 | 2.21E-13 |
|  |  | GO:0005178 | integrin binding | 14 | 5.42E-09 |
|  |  | GO:0005201 | extracellular matrix structural constituent | 10 | 3.23E-08 |
|  |  | GO:0030545 | signaling receptor regulator activity | 25 | 3.23E-08 |
|  |  | GO:0005539 | glycosaminoglycan binding | 17 | 3.23E-08 |
|  |  | GO:0048018 | receptor ligand activity | 23 | 1.56E-07 |
|  |  | GO:0030546 | signaling receptor activator activity | 23 | 1.56E-07 |
|  |  | GO:0005515 | protein binding | 121 | 1.56E-07 |
|  |  | GO:0044877 | protein-containing complex binding | 42 | 1.24E-06 |
|  |  | GO:0050839 | cell adhesion molecule binding | 16 | 2.17E-06 |
|  | KEGG | mmu04512 | ECM-receptor interaction | 8 | 0.008998 |
|  |  | mmu04020 | Calcium signaling pathway | 12 | 0.036489 |
|  |  | mmu04060 | Cytokine-cytokine receptor interaction | 12 | 0.053851 |
|  |  | mmu04151 | PI3K-Akt signaling pathway | 14 | 0.054491 |
|  |  | mmu05200 | Pathways in cancer | 18 | 0.055632 |
|  |  | mmu05142 | Chagas disease | 6 | 0.135297 |
|  |  | mmu04974 | Protein digestion and absorption | 6 | 0.140322 |
|  |  | mmu05205 | Proteoglycans in cancer | 8 | 0.193723 |
|  |  | mmu05206 | MicroRNAs in cancer | 7 | 0.198579 |
|  |  | mmu04915 | Estrogen signaling pathway | 6 | 0.199336 |
