# Supplementary material for: Identification and validation of SQLE in steroid-induced osteonecrosis of the femoral head: a bioinformatics and experimental study
Source: J Orthop Surg Res. 2025 Oct 17;20:894. doi: 10.1186/s13018-025-06305-x (PMC12533337; doi:10.1186/s13018-025-06305-x)
Supplement: Supplementary file 3 — Supplementary file3 (DOCX 19 kb) [file 13018_2025_6305_MOESM3_ESM.docx]

**Table S3.** **The ceRNA network.**

| **Node1** | **Node2** | **Interaction** |
| --- | --- | --- |
| SQLE | hsa-miR-877-3p | mRNA |
| SQLE | hsa-miR-205-5p | mRNA |
| LINC00689 | hsa-miR-877-3p | lncRNA |
| LINC00940 | hsa-miR-877-3p | lncRNA |
| FAR1-IT1 | hsa-miR-205-5p | lncRNA |
| RP1-253P7.1 | hsa-miR-877-3p | lncRNA |
| HMGCS2 | hsa-miR-29a-3p | mRNA |
| HMGCS2 | hsa-miR-624-3p | mRNA |
| RP11-333E1.2 | hsa-miR-624-3p | lncRNA |
| AC011718.2 | hsa-miR-624-3p | lncRNA |
| RP11-223P11.3 | hsa-miR-29a-3p | lncRNA |
| LSS | hsa-miR-335-5p | mRNA |
| LSS | hsa-miR-505-5p | mRNA |
| LSS | hsa-miR-640 | mRNA |
| LSS | hsa-miR-377-5p | mRNA |
| LSS | hsa-miR-877-3p | mRNA |
| LSS | hsa-miR-1207-5p | mRNA |
| LSS | hsa-miR-888-3p | mRNA |
| LSS | hsa-miR-875-3p | mRNA |
| LSS | hsa-miR-143-5p | mRNA |
| CTA-414D7.1 | hsa-miR-143-5p | lncRNA |
| CDR1-AS | hsa-miR-875-3p | lncRNA |
| CTC-265F19.1 | hsa-miR-143-5p | lncRNA |
| AC069257.8 | hsa-miR-640 | lncRNA |
| C10orf91 | hsa-miR-1207-5p | lncRNA |
| RP11-618K13.2 | hsa-miR-1207-5p | lncRNA |
| LINC00689 | hsa-miR-877-3p | lncRNA |
| LINC00265 | hsa-miR-1207-5p | lncRNA |
| RP11-333E1.2 | hsa-miR-1207-5p | lncRNA |
| LA16c-306A4.2 | hsa-miR-143-5p | lncRNA |
| CTB-51J22.1 | hsa-miR-505-5p | lncRNA |
| PCBP3-OT1 | hsa-miR-875-3p | lncRNA |
| AP001476.4 | hsa-miR-1207-5p | lncRNA |
| RP4-539M6.22 | hsa-miR-1207-5p | lncRNA |
| FAM95B1 | hsa-miR-888-3p | lncRNA |
| RP1-27K12.2 | hsa-miR-888-3p | lncRNA |
| LINC00940 | hsa-miR-877-3p | lncRNA |
| RP11-680F20.6 | hsa-miR-1207-5p | lncRNA |
| MUC19 | hsa-miR-640 | lncRNA |
| AC016682.1 | hsa-miR-505-5p | lncRNA |
| RP11-64K12.8 | hsa-miR-875-3p | lncRNA |
| RP11-867G23.4 | hsa-miR-1207-5p | lncRNA |
| FRMPD3-AS1 | hsa-miR-875-3p | lncRNA |
| LINC00686 | hsa-miR-143-5p | lncRNA |
| LINC00969 | hsa-miR-1207-5p | lncRNA |
| RP11-154H17.1 | hsa-miR-143-5p | lncRNA |
| H19 | hsa-miR-1207-5p | lncRNA |
| RP5-1142A6.2 | hsa-miR-1207-5p | lncRNA |
| CTD-2015G9.2 | hsa-miR-143-5p | lncRNA |
| LINC01168 | hsa-miR-1207-5p | lncRNA |
| AC000095.11 | hsa-miR-1207-5p | lncRNA |
| SLC8A1-AS1 | hsa-miR-335-5p | lncRNA |
| RP1-253P7.1 | hsa-miR-877-3p | lncRNA |
| MIRLET7BHG | hsa-miR-1207-5p | lncRNA |
| AP000442.1 | hsa-miR-377-5p | lncRNA |
| RP11-1030E3.1 | hsa-miR-335-5p | lncRNA |
| NSDHL | hsa-miR-484 | mRNA |
| NSDHL | hsa-miR-654-3p | mRNA |
| RP5-1092A3.5 | hsa-miR-484 | lncRNA |
| LINC00969 | hsa-miR-484 | lncRNA |
| LRRC75A-AS1 | hsa-miR-484 | lncRNA |
| FAM230B | hsa-miR-654-3p | lncRNA |
| AC078942.1 | hsa-miR-484 | lncRNA |
