# Supplementary material for: Identification and validation of SQLE in steroid-induced osteonecrosis of the femoral head: a bioinformatics and experimental study
Source: J Orthop Surg Res. 2025 Oct 17;20:894. doi: 10.1186/s13018-025-06305-x (PMC12533337; doi:10.1186/s13018-025-06305-x)
Supplement: Supplementary file 4 — Supplementary file4 (DOCX 17 kb) [file 13018_2025_6305_MOESM4_ESM.docx]

**Table S4.** **The drug-gene interactions network.**

| **Gene** | **Drug** | **Regulatory approval** | **Indication** | **Interaction score** |
| --- | --- | --- | --- | --- |
| SQLE | NAFTIFINE | Approved |  | 6.52547482 |
| SQLE | EPIGALLOCATECHIN GALLATE | Approved |  | 0.76770292 |
| SQLE | AMIODARONE HYDROCHLORIDE | Approved | antiarrhytmic agent | 1.003919203 |
| SQLE | TOLNAFTATE | Approved |  | 4.350316547 |
| FDPS | MINODRONIC ACID | Not Approved | antineoplastic agent | 2.175158273 |
| FDPS | IBANDRONATE | Approved | antiosteoporotic agent | 3.26273741 |
| FDPS | RIBOPRINE | Not Approved |  | 6.52547482 |
| FDPS | ETIDRONIC ACID | Approved |  | 0.233052672 |
| FDPS | ZOLEDRONIC ACID ANHYDROUS | Approved | antiosteoporotic agent | 1.522610791 |
| FDPS | TIPIFARNIB | Not Approved |  | 3.26273741 |
| FDPS | IBANDRONATE SODIUM | Approved |  | 3.26273741 |
| FDPS | RISEDRONIC ACID | Approved | Bisphosphonates,antiosteoporotic agent | 3.915284892 |
| FDPS | APLITHIANINE A | Not Approved |  | 0.652547482 |
| FDPS | INCADRONIC ACID | Not Approved |  | 1.631368705 |
| FDPS | NERIDRONIC ACID | Not Approved |  | 3.26273741 |
| FDPS | RISEDRONATE SODIUM ANHYDROUS | Approved |  | 3.26273741 |
| FDPS | PAMIDRONATE DISODIUM | Approved |  | 1.631368705 |
| FDPS | PAMIDRONATE | Approved | Bisphosphonates | 0.889837475 |
| FDPS | ALENDRONATE SODIUM | Approved | Bisphosphonates | 1.359473921 |
| FDPS | MINODRONIC ACID | Approved |  | 3.26273741 |
| HMGCS2 | HYDROCHLOROTHIAZIDE | Approved | antihypertensive agent | 1.134865186 |
| HMGCS2 | CHLOROTHIAZIDE | Approved | Antihypertensive Agents; Diuretics | 7.457685508 |
