# Supplementary material for: Identification and validation of SQLE in steroid-induced osteonecrosis of the femoral head: a bioinformatics and experimental study
Source: J Orthop Surg Res. 2025 Oct 17;20:894. doi: 10.1186/s13018-025-06305-x (PMC12533337; doi:10.1186/s13018-025-06305-x)
Supplement: Supplementary file 5 — Supplementary file5 (PDF 199 kb) [file 13018_2025_6305_MOESM5_ESM.pdf]

兰州大学实验动物中心伦理委员会

伦理审查批准

批准编号：MECI20250005

项目名称：脂代谢相关基因 SQLE 在股骨头坏死中的机制研究		
项目负责人：王文己	职称：副教授	联系电话：13893221698
研究单位：兰州大学第一医院		
研究项目来源：☐政府 ☐基金会 ☐公司 ☐国际合作 ✓自主 ☐其它		
请求审查类型：✓新申请项目 ☐修订后项目 ☐延续审查课题		

审查意见：

脂代谢相关基因 SQLE 在股骨头坏死中的机制研究 的研究方案和知情同意书经伦理委员会审查：

✓符合伦理学要求，同意按照此方案进行研究。

☐研究方案、☐知情同意书☐修改或☐补充资料后，伦理委员会同意开始研究。

☐不符合伦理要求，请修改后报伦理委员会再审查。

☐终止或☐暂停已批准的研究。

主任委员签（章）：[illegible]

兰州大学实验动物中心伦理委员会

（兰州大学实验动物中心代章）

2025 年 3 月 11 日
